# Metabolic engineering of stomatal precursor cells enhances photosynthetic water‐use efficiency and vegetative growth under water‐deficit conditions in *Arabidopsis thaliana*

**DOI:** 10.1111/pbi.70130

**Published:** 2025-05-23

**Authors:** Jacques W. Bouvier, Steven Kelly

**Affiliations:** ^1^ Department of Biology University of Oxford Oxford UK

**Keywords:** stomata, photosynthesis, water‐deficit, water‐use efficiency, growth, biomass

## Abstract

Stomata are epidermal pores that control the exchange of gaseous CO_2_ and H_2_O between plants and their environment. Modulating stomatal density can alter this exchange and thus presents a viable target for engineering improved crop productivity and climate resilience. Here, we show that stomatal density in *Arabidopsis thaliana* can be decreased by the expression of a water‐forming NAD(P)H oxidase targeted to stomatal precursor cells. We demonstrate that this reduction in stomatal density occurs irrespective of whether the expressed enzyme is localized to the cytosol, chloroplast stroma or chloroplast intermembrane space of these cells. We also reveal that this decrease in stomatal density occurs in the absence of any measurable impact on the efficiency and thermal sensitivity of photosynthesis, or on stomatal dynamics. Consequently, overexpression plants exhibit a higher intrinsic water‐use efficiency due to an increase in CO_2_ fixed per unit water transpired. Finally, we demonstrate that this enhanced water‐use efficiency translates to an improvement in vegetative growth and biomass accumulation under water‐deficit conditions. Together, these results thus provide a novel approach for enhancing plant productivity through metabolic engineering of stomatal density.

## Introduction

Stomata mediate the exchange of gaseous CO_2_ and water vapour between plants and their external environment. Specifically, CO_2_ provides the primary substrate of photosynthesis and its uptake is thereby required to fuel plant growth. In contrast, loss of water vapour is integral to the transpiration stream which helps regulate internal water status, temperature and nutrient uptake. As both CO_2_ and H_2_O gases share a single diffusion path but in opposite directions, controlling flux along this path is essential for balancing the physiological demands of the plant. Consequently, optimizing the distribution and behaviour of stomata is a central way in which plants adapt to different terrestrial habitats (Clark *et al*., 2022; Edwards *et al*., 1998; Raven, 2002).

Given their role at the interface between plants and their environment, stomata modulate leaf diffusive conductance in response to diverse stimuli including light (quality and quantity), humidity, temperature, soil‐water availability and atmospheric CO_2_ (Driesen *et al*., 2020). By integrating these signals alongside natural circadian rhythms, stomata open to promote CO_2_ fixation during the day and close at night or under adverse conditions to limit excessive water loss. In addition to this complex and dynamic behaviour, stomata also exhibit remarkable developmental plasticity across species and in response to diverse environmental changes. Most notably, stomatal density inversely correlates with soil‐water availability (Quarrie and Jones, 1977), temperature (Lau *et al*., 2018) and ambient CO_2_ concentration (Beerling *et al*., 1998, 2001; Franks and Beerling, 2009; McElwain and Chaloner, 1995; Woodward, 1987; Woodward *et al*., 2002; Woodward and Bazzaz, 1988) and positively correlates with relative air humidity (Bakker, 1991; Fanourakis *et al*., 2013) over developmental or geological timescales, respectively. These environmentally induced changes in density are generally linked with secondary adjustments in stomatal size (such that higher densities of stomata are generally smaller in size and *vice versa* (Buckley *et al*., 2020; de Boer *et al*., 2016; Dittberner *et al*., 2018; Doheny‐Adams *et al*., 2012)), though density always takes precedence over size as the predominant anatomical constraint on leaf gaseous conductance (Mcelwain *et al*., 2016; McElwain and Chaloner, 1995). Thus, both dynamic and developmental changes in stomata play important roles in the growth and environmental adaptation of plants.

The considerable plasticity of stomatal form and function, coupled with the importance of stomata in regulating plant growth and environmental interactions, have together inspired multiple attempts to alter their properties for crop improvement. Pioneering efforts of Farquhar and Richards in the 1980's used stable carbon isotope ratios to screen for enhanced water‐use efficiency in wheat (Condon *et al*., 2002; Farquhar and Richards, 1984). Subsequently, targeted engineering efforts have included a range of forward genetic approaches which have manipulated the distribution of stomata (Dow *et al*., 2014; Nadeau and Sack, 2002; Papanatsiou *et al*., 2016; Shpak *et al*., 2005; Wang *et al*., 2007; Yu *et al*., 2020), as well as the mechanical (Carroll *et al*., 2022), transport (Antunes *et al*., 2017; Flütsch *et al*., 2020; Horaruang *et al*., 2022; Huang *et al*., 2021; Kusumi *et al*., 2012; Medeiros *et al*., 2016, 2017; Papanatsiou *et al*., 2019; Reyer *et al*., 2020; Toh *et al*., 2021; Wang *et al*., 2014, 2019; Zhou *et al*., 2021) and metabolic (Acevedo‐Siaca *et al*., 2022; Antunes *et al*., 2017, 2012; Boxall *et al*., 2020; Daloso *et al*., 2016; Flütsch *et al*., 2020; Horrer *et al*., 2016; Kelly *et al*., 2012, 2019; Laporte *et al*., 2002; Lugassi *et al*., 2015, 2019; Müller *et al*., 2018; Penfield *et al*., 2012; Prasch *et al*., 2015) properties of guard cells. Of all these approaches, altering stomatal density has received the most attention, and a vast array of mutants with both increased (Bergmann *et al*., 2004; Bussis *et al*., 2006; Franks *et al*., 2015; Gudesblat *et al*., 2012; Hepworth *et al*., 2015; Jalakas *et al*., 2018; Masle *et al*., 2005; Pillitteri *et al*., 2007; Sakoda *et al*., 2020; Schlüter *et al*., 2003; Schuler *et al*., 2018; Tanaka *et al*., 2013; Yang and Sack, 1995) and decreased (Bergmann *et al*., 2004; Bussis *et al*., 2006; Caine *et al*., 2019; Doheny‐Adams *et al*., 2012; Dunn *et al*., 2019; Franks *et al*., 2015; Gudesblat *et al*., 2012; Hara *et al*., 2009, 2007; Hepworth *et al*., 2015; Hughes *et al*., 2017; Jalakas *et al*., 2018; Mohammed *et al*., 2019; Pillitteri *et al*., 2007; Wang *et al*., 2016; Yin *et al*., 2017; Yoo *et al*., 2010; Zhao *et al*., 2024) stomatal densities have been engineered across diverse plant and crop species.

A common theme from approaches that have altered stomatal densities has been the genetic manipulation of the expression levels of stomatal development genes. Such targets have included the basic helix–loop–helix transcription factors SPEECHLESS (SPCH) (Gudesblat *et al*., 2012), MUTE (Pillitteri *et al*., 2007) and FAMA (Bergmann *et al*., 2004) which regulate the initiation, proliferation and differentiation stages of the stomatal lineage, respectively. Engineering success has also been achieved by altering the expression levels of other important components of stomatal development, including the EPIDERMAL PATTERNING FACTOR family of signalling peptides (Caine *et al*., 2019; Doheny‐Adams *et al*., 2012; Dunn *et al*., 2019; Franks *et al*., 2015; Hara *et al*., 2009, 2007; Hepworth *et al*., 2015; Hughes *et al*., 2017; Mohammed *et al*., 2019; Sakoda *et al*., 2020; Tanaka *et al*., 2013; Wang *et al*., 2016; Yin *et al*., 2017) alongside their receptor components (TOO MANY MOUTHS (Yang and Sack, 1995) and the ERECTA protein kinases (Masle *et al*., 2005)), STOMATAL DENSITY AND DISTRIBUTION 1 (SDD1) and its respective interactor components (Bussis *et al*., 2006; Schlüter *et al*., 2003; Yoo *et al*., 2010), as well as a host of other genes involved in hormone signalling and non‐canonical stomatal developmental pathways (Jalakas *et al*., 2018; Schuler *et al*., 2018; Zhao *et al*., 2024). However, despite these considerable successes, the molecular and biochemical mechanisms that link these gene networks to changes in environmental and physiological cues remain poorly understood.

Nicotinamide adenine dinucleotide (NAD^+^) presents a potential metabolic nexus that can link physiological changes in the leaf to the genetic regulation of stomatal patterning (de Souza Chaves *et al*., 2019; Falquetto‐Gomes *et al*., 2024; Feitosa‐Araujo *et al*., 2020). Recent studies provide evidence for this potential role by demonstrating that decreases in stomatal densities are produced in *Arabidopsis thaliana* from changes in cellular NAD^+^ status associated with either exogenous NAD^+^ treatment (Feitosa‐Araujo *et al*., 2020), knockdown of mitochondrial (NDT1 and NDT2) and peroxisomal (PXN1) NAD^+^ transporters (de Souza Chaves *et al*., 2019; Feitosa‐Araujo *et al*., 2020) or knockdown of poly(ADP‐ribose)polymerase genes involved in NAD^+^ recycling (Feitosa‐Araujo *et al*., 2020). This NAD^+^‐mediated alteration in stomatal patterning is thought to occur via perturbation of abscisic acid (ABA) biosynthesis and signalling, which suppresses the activity of SPCH (Yang *et al*., 2022) and in turn acts to inhibit cell entry into the stomatal lineage (Chater *et al*., 2015, 2014; Lake and Woodward, 2008; Quarrie and Jones, 1977; Tanaka *et al*., 2013). This connection between NAD^+^ and ABA was postulated because changes in NAD^+^ homeostasis were associated with both transcriptional changes in ABA metabolic genes and endogenous levels of this phytohormone, and because alterations in stomatal density in mutants could be rescued by inhibition of ABA biosynthesis (Feitosa‐Araujo *et al*., 2020). Intriguingly, this also aligns with the known interaction of both NAD^+^ and ABA in regulating stomatal aperture (Hashida *et al*., 2010; Sun *et al*., 2017). Thus, as NAD^+^ is one of the cornerstones of plant metabolism, the recent discovery that it also has a role in regulating stomatal cell differentiation provides a simple and unified route through which changes in physiology or environment directly feedback on stomatal patterning.

Water‐forming NAD(P)H oxidases (NOX) present a powerful genetically‐encoded tool for studying the role of redox status on organismal developmental processes. Specifically, NOX are a class of enzymes which catalyse the four‐electron reduction of molecular oxygen in the presence of protons and reducing equivalents NADH and nicotinamide adenine dinucleotide phosphate (NADPH) to produce water and the corresponding NAD^+^/NADP^+^ electron acceptor in the following stoichiometry [2NAD(P)H + 4H^+^ + O_2_ → 2NAD(P)^+^ + 2H_2_O]. In nature, these enzymes are thought to function in both oxidative stress management as well as in balancing the relative levels of oxidized and reduced forms of co‐factor pools (Yan *et al*., 2016). Due to this physiological role and the fact that NOX enzymes are highly catalytically efficient, they have been exploited to alter ratios of oxidized and reduced co‐factor pools in diverse fundamental and applied science contexts (Cracan *et al*., 2017; de Gonzalo *et al*., 2012; Eiteman *et al*., 2006; Heux *et al*., 2006; Kroutil *et al*., 2004; Titov *et al*., 2016; Turner, 2011; Vemuri *et al*., 2007).

In this study, we aimed to engineer stomatal density through NOX‐mediated manipulation of NAD(P)^+^/NAD(P)H status in stomatal precursor cells. We show that active monomeric NOX can be targeted to different compartments in plant cells. We further show that the expression of NOX across these diverse subcellular compartments of stomatal precursor cells leads to a reduction in stomatal density without any adverse effects on stomatal function or leaf‐level photosynthesis. Although maximal rates of CO_2_ assimilation are not affected in transgenic plants, the intrinsic photosynthetic wateruse efficiency is improved such that more CO_2_ is fixed per unit water transpired by the leaf. Accordingly, this results in enhanced growth and biomass accumulation under water‐deficit conditions, whilst maintaining wild‐type levels of growth under well‐watered conditions. In summary, this study thus provides a novel and tractable metabolic engineering approach to manipulate stomatal patterning as a potential target to enhance crop productivity and climate resilience.

## Results

### NOX can be expressed in plant cells and targeted to multiple subcellular locations

Diverse water‐forming NAD(P)H oxidases (NOX) enzymes have been characterized across bacteria (Cracan *et al*., 2017; Dishisha *et al*., 2019; Gao *et al*., 2019, 2012; Geueke *et al*., 2003; Higuchi *et al*., 1993; Hummel and Riebel, 2003; Kawasaki *et al*., 2004; Li *et al*., 2019; Lopez de Felipe and Hugenholtz, 2001; Lountos *et al*., 2006; Matsumoto *et al*., 1996; Nowak *et al*., 2015; Park *et al*., 2011; Riebel *et al*., 2002; Sakamoto *et al*., 1996; Schmidt *et al*., 1986; Stanton and Jensen, 1993; Titov *et al*., 2016; Wang *et al*., 2012; Zhang *et al*., 2012), archaea (Jia *et al*., 2008, 2010; Nisar *et al*., 2013; Rashid *et al*., 2011; Ward *et al*., 2001; Wu *et al*., 2012; Yan *et al*., 2016) and eukaryotes (Brown *et al*., 1996; Castillo‐Villanueva *et al*., 2016; Li and Wang, 2006). Here, we used an engineered variant of this enzyme from the bacterium *Streptococcus mutans* (hereinafter, termed *Sm*NOX) as the basis of all experiments (Petschacher *et al*., 2014). This variant was chosen based on several criteria. First, *Sm*NOX exhibits NAD(P)H bifunctionality (Petschacher *et al*., 2014) and is thus capable of oxidizing co‐factor pools in a range of eukaryotic cellular contexts. Second, *Sm*NOX is functional across a broad pH and temperature range with optimal activities matching physiological conditions *in planta* (Higuchi *et al*., 1993; Li *et al*., 2018). Third, *Sm*NOX is monomeric (Higuchi *et al*., 1993) allowing a higher possibility for it to tolerate protein–protein fusions and N‐terminal targeting peptides whilst maintaining functional activity. Finally, *Sm*NOX exhibits negligible secondary H_2_O_2_ production that may contribute to undesirable off‐target effects (Li *et al*., 2018; Petschacher *et al*., 2014).

**Figure 1:**
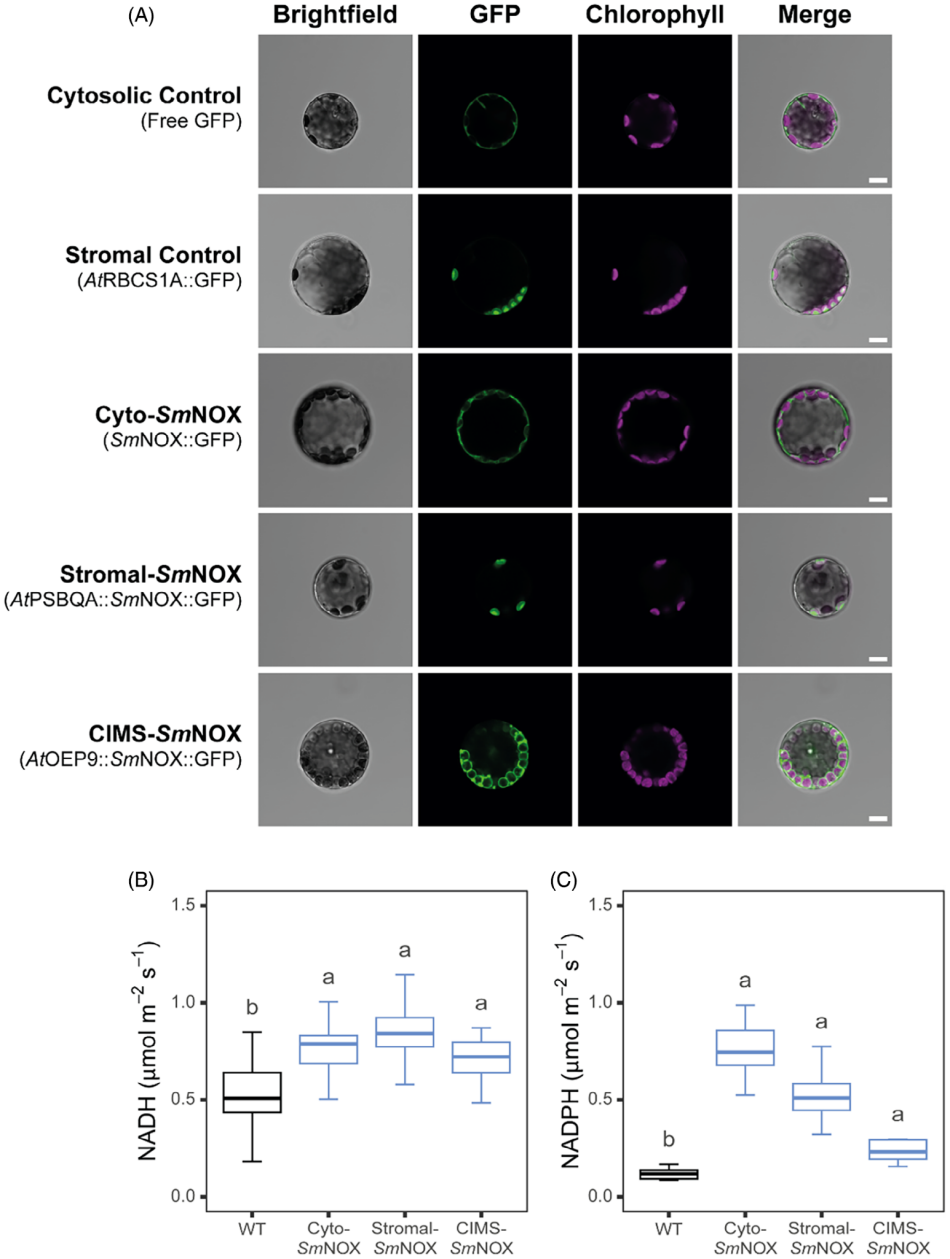
Validation of *Sm*NOX localisation and functional activity *in planta*. (A) The subcellular localisation of *Sm*NOX in *Arabidopsis thaliana* protoplasts. Free GFP: protoplasts expressing eGFP (cytosolic control). *At*RBCS1A::GFP: protoplasts expressing eGFP fused at the N‐terminus to the rubisco small subunit (chloroplast stromal control). Cyto‐*Sm*NOX: protoplasts expressing *Sm*NOX fused at the C‐terminus to eGFP (cytosolic localisation). Stromal‐*SmNOX*: protoplasts expressing *SmNOX* fused at the C‐terminus to eGFP and fused at the N‐terminus to the chloroplast transit peptide of the photosystem II subunit QA protein (chloroplast stromal localisation). CIMS‐*Sm*NOX: protoplasts expressing *SmNOX* fused at the C‐terminus to eGFP and fused at the N‐terminus to the full‐length outer envelope protein 9 (CIMS; chloroplast intermembrane space localisation). Three separate signals are shown. Green: eGFP fluorescence. Violet: Thylakoid‐derived chlorophyll autofluorescence. Grey: bright‐field. The merged channel represents all three signals overlaid in a single image. Scale bar = 10 μm. (B) Boxplot depicting the rate of NADH oxidation from crude leaf lysate of wild‐type (black) and transgenic plants (blue). Abbreviations follow that described in (A). Data represent an average across three independent single copy lines (*n* = 4 plants per line). (C) As in (B), but for the total NADPH oxidation from crude leaf lysate (*n* = 5 plants per line). Differences between transgenic plants and WT are assessed by Fisher LSD *post‐hoc* analysis following a two‐way ANOVA, where letters above each box represent statistically significant differences in mean values (*P* ≤ 0.05). The raw data can be found in Appendix S4.

To assess whether NOX can be produced in plants, we first tested *Sm*NOX expression using a transient protoplast expression system. For this purpose, mesophyll protoplasts were isolated from mature rosette leaves of *A. thaliana* and transformed with a genetic construct containing the full‐length *Sm*NOX coding sequence translationally fused to a C‐terminal GFP (Appendix S1, Figure S1A). Analysis of cell fluorescence using confocal microscopy confirmed that the *Sm*NOX‐GFP fusion was translated in plant cells and that the enzyme localized to the cytosol when driven in the absence of any additional targeting sequences (Figure 1A), in agreement with a predicted bioinformatic localization (Appendix S1, Table S1).

Whilst NAD^+^ is produced in the cytosol, the regulatory mechanism that governs cytosolic NAD^+^ levels is dependent on chloroplast NADP^+^ production. Accordingly, we sought to test whether *Sm*NOX could also be localized closer to the site of NADP^+^ supply in both the chloroplast stroma (the site of NADPH production) and the chloroplast intermembrane space (the site of NADPH/NADH transfer via the malate valve). *Sm*NOX was successfully localized to the chloroplast stroma (stromal‐*Sm*NOX) when fused to the transit peptide from the photosystem II subunit QA (PSBQA) protein (Figure 1A). This stromal localisation was verified by comparison to a stromal control consisting of the full‐length rubisco small subunit gene fused to GFP (Figure 1A). Moreover, *Sm*NOX was also successfully targeted to the chloroplast intermembrane space (CIMS‐*Sm*NOX) when fused to the full‐length outer envelope protein 9 gene (Figure 1A). In summary, *Sm*NOX can thus be expressed in plants and can be localized to diverse subcellular compartments including the cytosol (the site of NAD^+^ production), chloroplast stroma (the site of NADP^+^ production) and chloroplast intermembrane space (the site of NAD^+^/NADP^+^ transfer).

### Identifying a promoter capable of driving stable and high expression of *Sm*NOX in stomatal precursor cells

To investigate whether *Sm*NOX could affect plant stomatal patterning, it was necessary to next find a promoter which drives high gene expression in undifferentiated stomatal progenitor cells in the leaf epidermis (i.e., during the developmental window prior to onset of stomatal lineage differentiation) (Appendix S1, Figure S2A). For this purpose, we analysed a cell‐specific transcriptome dataset derived from all major cell types spanning the stomatal developmental trajectory (Adrian *et al*., 2015). *A priori*, we hypothesized that promoters from the transcription factors SPEECHLESS (SPCH), MUTE and FAMA might provide promising candidates for driving transgene expression in stomatal precursor cells given their roles in co‐ordinating stomatal development. However, interrogation of the transcriptome dataset revealed that the genes encoding SPCH, MUTE and FAMA were all expressed at low levels which were considered to be insufficient to achieve *Sm*NOX‐mediated alteration of cellular NAD^+^ status (Appendix S1, Figure S2B–D). Consequently, we sought to identify an alternative, more highly expressed promoter.

Interrogation of the above transcriptome data revealed that the gene encoding the chlorophyll *a*/*b* binding protein 3 (CAB3) exhibited high mRNA abundance in the target epidermal tissue of interest (Appendix S1, Figure S2B). Specifically, CAB3 was ranked as the 36^th^ (out of ~28 000) most highly expressed nuclear‐encoded gene in this cell type (Appendix S1, Figure S2C,D) and exhibited transcript levels that were ~ 1800‐fold more abundant than SPCH (0.5 TPM), ~9000‐fold more abundant than MUTE (0.1 TPM) and ~425‐fold more abundant than FAMA (2.1 TPM), respectively (Appendix S1, Figure S2B–D). Thus, as CAB3 is one of the most abundant transcripts in the cell type of interest, and as CAB3 is a well‐characterized promoter that is extensively used for driving transgene expression in plant synthetic biology (Mitra *et al*., 1989), this promoter was selected for use herein. Genetic constructs containing targeted and untargeted variants of the *Sm*NOX coding sequence were thus cloned under the control of the CAB3 promoter and were transformed into *A. thaliana* ecotype Col‐0 (Appendix S1, Figures S1B and S3A,B). Homozygous plants from three single‐copy insertion events of cyto‐*Sm*NOX, stromal‐*Sm*NOX and CIMS‐*Sm*NOX were used for the basis of all subsequent experiments.

### Validation of *Sm*NOX expression and function

To assess the functional activity of *Sm*NOX in plants, total rates of NADH and NADPH oxidation were determined using *in vitro* assays containing crude lysate from mature rosette leaves. As plants possess multiple native enzymes capable of oxidizing NADH and NADPH, background oxidation of NADH and NADPH was observed in all cases as expected (Figure 1B,C). However, the rates of both NADH and NADPH oxidation were significantly increased across all transgenic lines compared to wild‐type controls (Figure 1B,C). Thus, there was successful expression of active *Sm*NOX in each of the targeted subcellular localisations.

It should be noted that the CAB3 promoter also drives high levels of transgene expression in other photosynthetic cells of the mature leaf (Appendix S1, Figures S2B–D and S4A–C). Thus, although our target tissue for expression of *Sm*NOX is the leaf epidermis, only a portion of the above enzyme activity in the crude leaf lysate corresponds to this cell layer. Moreover, whilst the measured rates of *Sm*NOX‐mediated NADH/NADPH oxidation (~1.0 μmol/m^2^/s) are substantial in the context of an epidermal cell, they are not expected to impact NADH/NADPH pools in photosynthetic mesophyll cells. This is because of both the large pool size of NADH (24–55 μm) and NADPH (140–180 μm) (Igamberdiev and Gardeström, 2003) and the extraordinarily high rates of photosynthetic NADPH production (~70 μmol/g/s) (Wieloch and Sharkey, 2022) in the mesophyll tissue. Thus, we hypothesized that *Sm*NOX would be capable of manipulating endogenous levels of NAD^+^ in leaf cells with low photosynthetic activity such as epidermal cells, but would have little or no effect on the NAD^+^ status or photosynthesis of mesophyll cells.

### *Sm*NOX activity causes reductions in stomatal density

**Figure 2:**
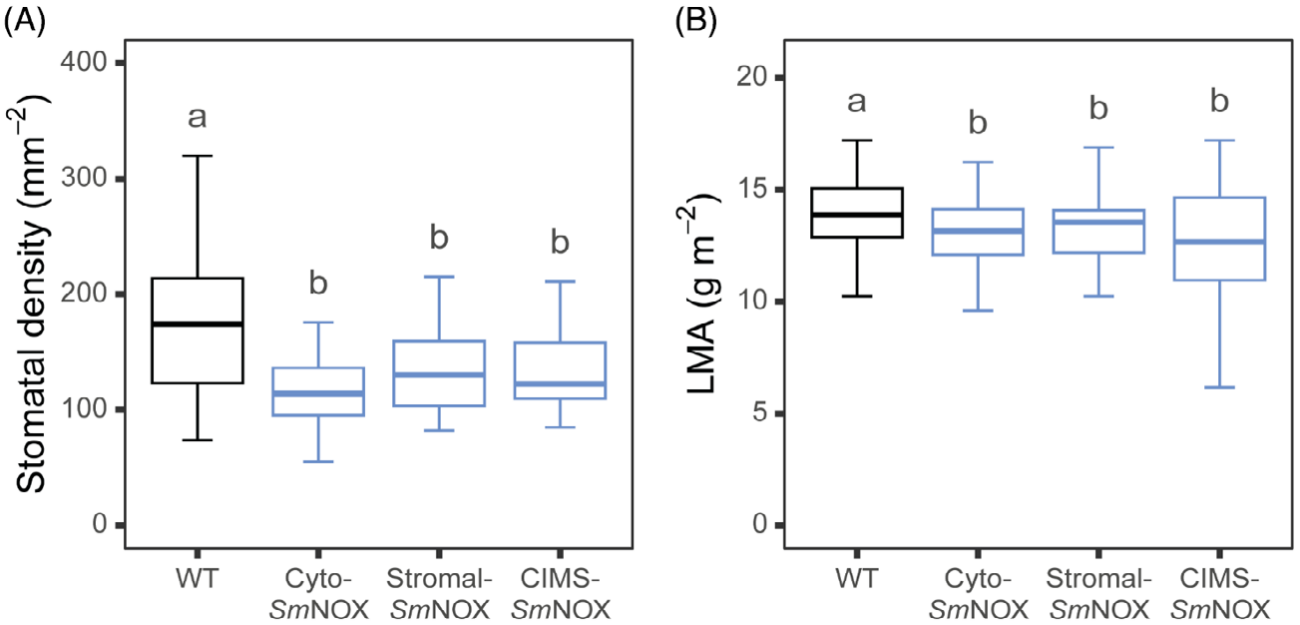
The effect of *Sm*NOX expression on stomatal density. (A) Boxplot depicting the stomatal density (count/mm^2^) measured on the abaxial surface of mature rosette leaves from wild‐type (black) and transgenic plants (blue). WT: wild‐type. Cyto‐*Sm*NOX: cytosolic *Sm*NOX. Stromal‐*Sm*NOX: chloroplast stromal *Sm*NOX. CIMS‐*Sm*NOX: chloroplast intermembrane space *Sm*NOX. Data represent an average across three independent single copy lines (*n* = 9–10 plants per line). (B) As in (A), but for the measured leaf mass per area (LMA, g dry mass/m^2^ leaf area) of mature rosette leaves (*n* = 10 plants per line). Differences between transgenic plants and WT are assessed by Fisher LSD *post‐hoc* analysis following a two‐way ANOVA, where letters above each box represent statistically significant differences in mean values (*P* ≤ 0.05). The raw data can be found in Appendix S4.

Given that *Sm*NOX was active in transgenic plants, we next investigated whether this resulted in an alteration in stomatal patterning. For this purpose, the epidermal composition of the abaxial surface of mature rosette leaves from 8‐week‐old plants was analysed by bright‐field microscopy. This analysis revealed that stomatal densities in all *Sm*NOX‐expressing lines were reduced by 20%–30% compared to wild‐type control plants (Figure 2A). Thus, changes in cellular NAD(P)^+^/NAD(P)H ratios, irrespective of the location of perturbation of that ratio, caused a suppression in stomatal development resulting in a reduced density of stomata on leaves.

Stomatal density changes have previously been linked with secondary changes in mesophyll development (such that leaves with reduced stomatal numbers are generally thinner and *vice versa* (Dow *et al*., 2017; Pérez‐Bueno *et al*., 2022)) which can have important consequences for plant physiology (Baillie and Fleming, 2020; Bergmann, 2004; Kondo *et al*., 2010; Lundgren *et al*., 2019; Savaldi‐Goldstein *et al*., 2007; Serna and Fenoll, 2000; Vile *et al*., 2012). Thus, we also investigated whether *Sm*NOX expression was associated with an alteration in leaf width or cellular density by measuring leaf mass per area (LMA = leaf dry mass/leaf area). This revealed *Sm*NOX‐expressing lines exhibited a small but significant reduction in LMA compared to wild‐type (Figure 2B). Thus, the expression of *Sm*NOX results in both a decrease in stomatal patterning and anticipated secondary alterations in leaf architecture.

### *Sm*NOX‐induced changes in stomatal density improve plant photosynthetic water‐use efficiency

**Figure 3:**
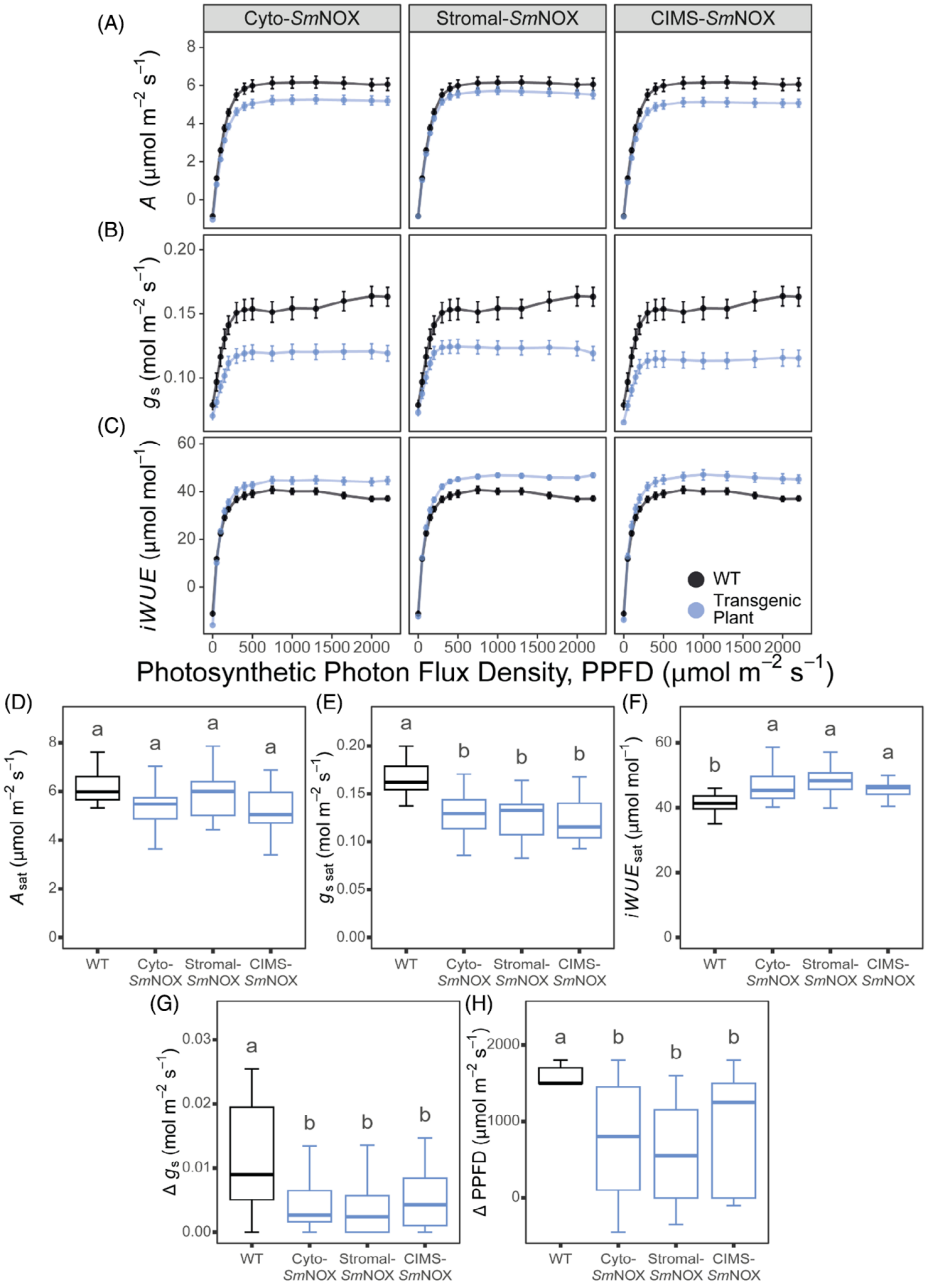
The response of plant gas exchange to light. (A) CO_2_ assimilation rate (*A*, μmol/m^2^/s) at different PPFD in wild‐type (black) and transgenic plants (blue). WT: wild‐type. Cyto‐*Sm*NOX: cytosolic *Sm*NOX. Stromal‐*Sm*NOX: chloroplast stromal *Sm*NOX. CIMS‐*Sm*NOX: chloroplast intermembrane space *Sm*NOX. Data represent mean ± 1 SE. (B) As in (A), but for stomatal conductance (*g*_s_, mol/m^2^/s). (C) As in (A), but for intrinsic water‐use efficiency (*iWUE*, μmol/mol). (D) Boxplot depicting the light‐saturated assimilation rate (*A*_sat_, μmol/m^2^/s). The colour scheme and abbreviations follow that described in (A). (E) As in (D), but for the light‐saturated stomatal conductance (*g*_s sat_, mol/m^2^/s). (F) As in (D), but for the light‐saturated intrinsic water‐use efficiency (*iWUE*_sat_, μmol/mol). (G) Boxplot depicting the difference in stomatal conductance between the light intensity at which 95% maximum assimilation rate is achieved and the light‐saturated stomatal conductance rate, respectively (Δ *g*_s_, mol/m^2^/s). (H) As in (G), but for the difference in light intensity at which 95% maximum assimilation rate and the light‐saturated stomatal conductance are achieved (Δ PPFD, μmol/m^2^/s). All data represent an average across three independent single copy lines (*n* = 7–8 plants line). Differences between transgenic plants and WT are assessed by Fisher LSD *post‐hoc* analysis following a two‐way ANOVA, where letters above each box represent statistically significant differences in mean values (*P* ≤ 0.05). The raw data can be found in Appendix S4.

As stomatal density is a key determinant of leaf gaseous conductance, we next characterized the effect of *Sm*NOX expression on plant physiological performance. To test this, we first assessed the photosynthetic parameters of *Sm*NOX‐expressing lines along a light intensity gradient using an infrared open‐gas exchange system (Figure 3A–C). This analysis revealed that there was a small reduction in CO_2_ assimilation rate (*A*) between *Sm*NOX‐expressing lines and wild‐type plants (Figure 3A). However, stomatal conductance (*g*_s_) was substantially reduced across all *Sm*NOX‐expressing lines (Figure 3B), consistent with these transgenic plants exhibiting lower ratios of intercellular CO_2_ to ambient CO_2_ (*C*_i_/*C*_a_) and reduced rates of transpiratory water loss under high light (Appendix S1, Figure S5A,B). In line with these results, *Sm*NOX‐expressing lines exhibited broadly comparable light‐saturated rates of CO_2_ assimilation (*A*_sat_, Figure 3D, Appendix S1, Table S2) whilst maintaining substantially lower light‐saturated rates of stomatal conductance (*g*_s sat_, Figure 3E, Appendix S1, Table S2). As a consequence of these combined effects on *A* and *g*_s_, all *Sm*NOX‐expressing lines exhibited a higher intrinsic water‐use efficiency (*iWUE = A*/*g*_*s*_) across a broad range of light intensities (Figure 3C,F, Appendix S1, Table S2).

In addition to an enhanced *iWUE*, *Sm*NOX expression also resulted in tighter coordination between rates of *A* and *g*_s_. Specifically, all mutants displayed a reduced “overshooting” of *g*_s_ at light intensities above those where 95% maximum assimilation was achieved (beyond which neither CO_2_ delivery nor light is considered limiting) (Appendix S1; Figure 3G,H; Appendix S1, Figure S6A–F, Table S3). This improved *iWUE* and tighter coupling between *A* and *g*_s_ was not associated with a change in any other measured photosynthetic parameter including light compensation point, mitochondrial respiration in the light, apparent quantum yield (i.e., the efficiency by which light can be converted into ATP and NADPH) or the light‐saturated rate of electron transport (Appendix S1, Figure S5C–F, Table S2). Moreover, there was no consistent difference in the response of CO_2_ assimilation as a function of sub‐stomatal CO_2_ concentration (Appendix S1, Figure S7A), or any derived photosynthetic parameter computed from this analysis (Appendix S1, Figure S7B–G, Table S4). Specifically, *Sm*NOX expression did not result in any change in the maximal CO_2_ saturated assimilation rate, CO_2_‐compensation point, the transition point between rubisco‐limited and RuBP regeneration‐limited rates of photosynthesis, the rubisco Michaelis constant for CO_2_ in the presence of 21% O_2_ air, the carboxylation rate of rubisco or the maximum electron transport rate through PSII (Appendix S1, Figure S7B–G, Table S4). Importantly, there was also no change between wild‐type and *Sm*NOX‐expressing lines in the measured stomatal limitation on plant CO_2_ assimilation rate (Appendix S1, Figure S8A–C, Table S5). As anticipated, NOX activity was thus able to alter stomatal density, but was not sufficient to exert any measurable impact on mesophyll cell photosynthesis. Accordingly, *Sm*NOX‐mediated reductions in stomatal density drives both an enhanced photosynthetic water‐use efficiency and a tighter coordination between rates of CO_2_ assimilation and gas exchange.

### *Sm*NOX does not affect the kinetics of stomatal movement

We next sought to examine whether changes in stomatal density caused by *Sm*NOX expression were also associated with off‐target impacts on stomatal behaviour. Specifically, we chose to assess the speed and magnitude of stomatal responses to light, given that this is one of the most important fluctuating variables that plants face under real‐world conditions in the field. Light was also chosen because NADP^+^ is the terminal electron acceptor in the photosynthetic electron transport chain, and thus the impact of light on stomatal kinetics had the potential to be perturbed by *Sm*NOX expression. For the purpose of this analysis, the experimental design of (McAusland *et al*., 2016) was followed. In brief, plants were first acclimated at 100 μmol/m^2^/s of light for 60 min, followed by a step increase in light intensity to 1000 μmol/m^2^/s for 60 min and a final step decrease in light intensity to 100 μmol/m^2^/s for 30 min, with gas exchange parameters recorded at 1‐min intervals.

**Figure 4:**
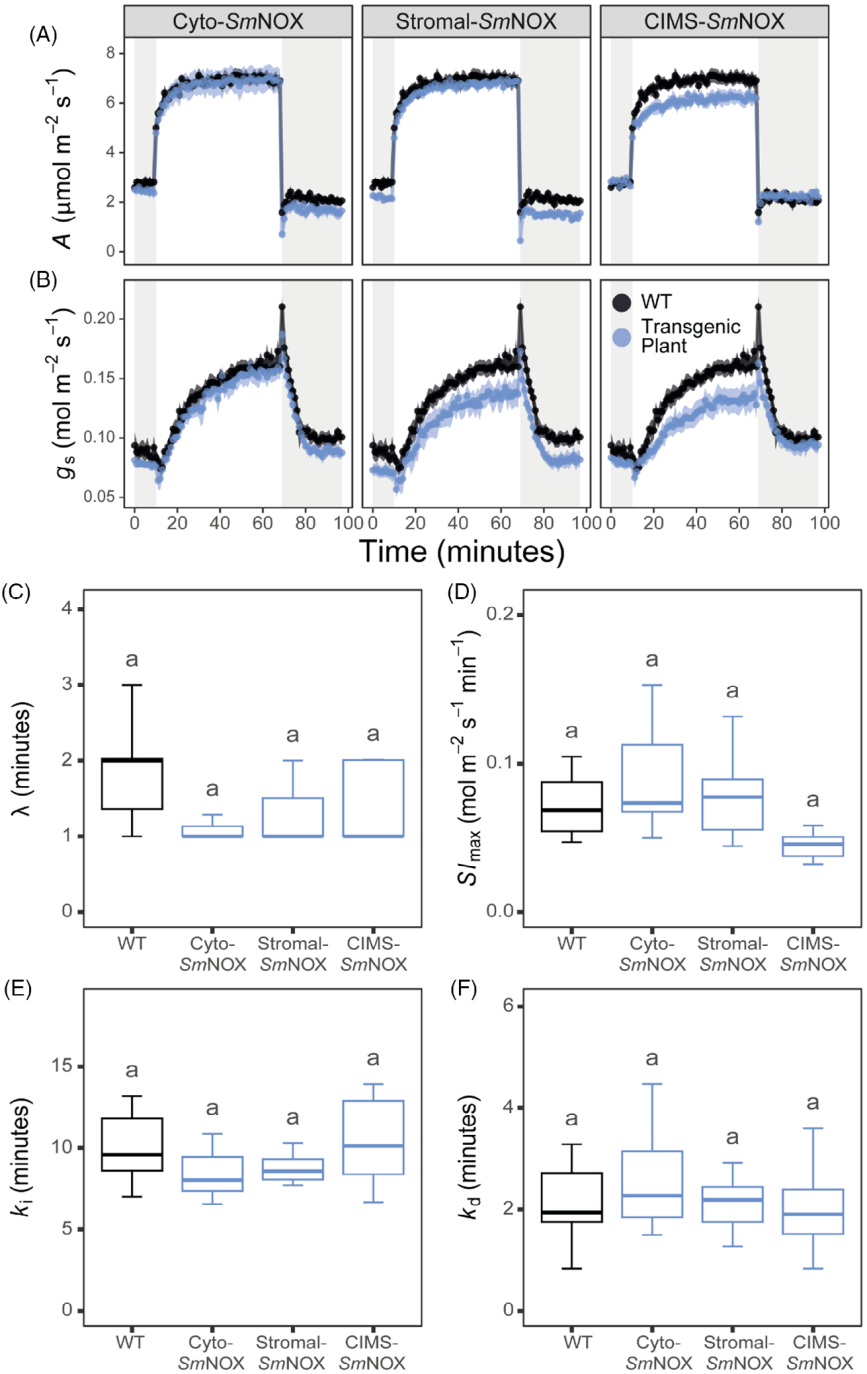
The temporal response of plant gas exchange to step changes in light intensity. (A) The response of CO_2_ assimilation rate (*A*, μmol/m^2^/s) to step changes in light intensity in wild‐type (black) and transgenic plants (blue). Shaded and unshaded areas represent periods of light intensity of 100 and 1000 μmol/m^2^/s, respectively. WT: wild‐type. Cyto‐*Sm*NOX: cytosolic *Sm*NOX. Stromal‐*Sm*NOX: chloroplast stromal *Sm*NOX. CIMS‐*Sm*NOX: chloroplast intermembrane space *Sm*NOX. Data represent mean ± 1 SE. (B) As in (A), but for stomatal conductance (*g*_s_, mol/m^2^/s). (C) Boxplot depicting the estimated initial lag in response time of *g*_s_ (λ, minutes) after the step increase in light intensity from 100 to 1000 μmol/m^2^/s. The colour scheme and abbreviations follow that described in (A). (D) As in (C), but for the estimated maximal rate of stomatal opening (*Sl*_max_, mol/m^2^/s/min) after the step increase in light intensity from 100 to 1000 μmol/m^2^/s. (E) As in (C), but for the estimated time taken to achieve new steady state stomatal conductance (*k*_i_, minutes) after the step increase in light intensity from 100 to 1000 μmol/m^2^/s. (F) As in (C), but for the estimated time taken to achieve new steady state stomatal conductance (*k*_d_, minutes) after the step decrease in light intensity from 1000 to 100 μmol/m^2^/s. All data represent an average across three independent single copy lines (*n* = 2–4 plants per line). Differences between transgenic plants and WT are assessed by Fisher LSD *post‐hoc* analysis following a two‐way ANOVA, where letters above each box represent statistically significant differences in mean values (*P* ≤ 0.05). The raw data can be found in Appendix S4.

This analysis revealed that all plants experience a typical profile of both *A* and *g*_s_ in response to step changes in irradiance (Figure 4A,B, Appendix S1, Figure S9). Moreover, subsequent model‐based interrogation (Vialet‐Chabrand *et al*., 2013) of these *g*_s_ data demonstrated that neither the amplitude nor the speed of stomatal responses was different between wild‐type and transgenic plants. Specifically, no difference was found in either the modelled initial lag time in *g*_s_, the maximum rate of change of *g*_s_ (calculated from the slope of the exponential phase of the curve) or the time taken for *g*_s_ to reach new steady state upon the initial step increase in light intensity from 100 to 1000 μmol/m^2^/s (Figure 4C–E, Appendix S1, Table S6). Similarly, no difference could be detected in the time taken to reach new steady state *g*_s_ upon the subsequent step decrease in light intensity from 1000 to 100 μmol/m^2^/s (Figure 4F, Appendix S1, Table S6). Thus, consistent with the lack of effect of *Sm*NOX on plant photosynthesis, *Sm*NOX expression did not impair the dynamic behaviour of stomatal movement.

### *Sm*NOX does not alter plant susceptibility to supra‐optimal temperatures

**Figure 5:**
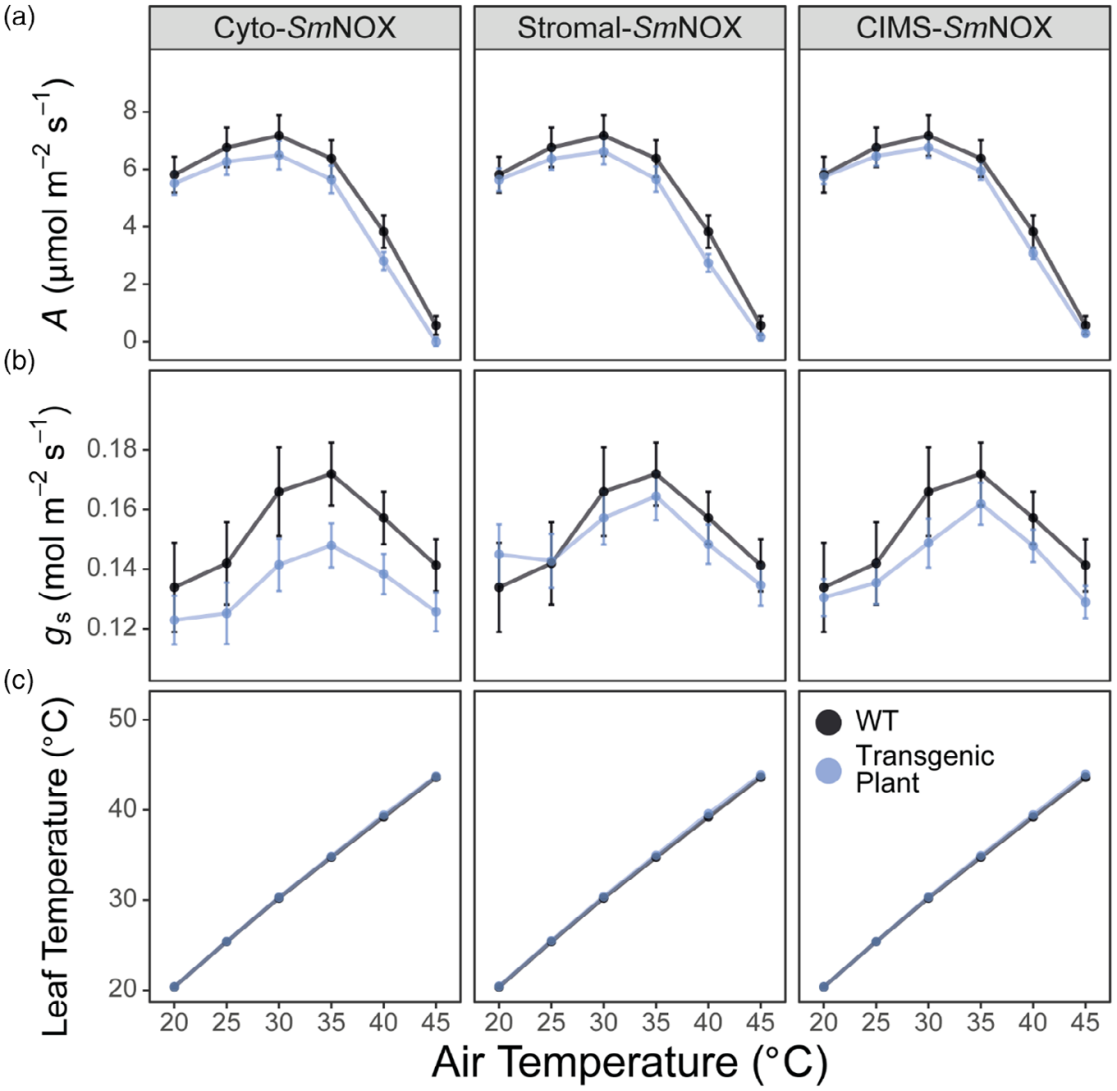
The response of plant gas exchange to air temperature (°C). (a) CO_2_ assimilation rate (*A*, μmol/m^2^/s) at different air temperatures in wild‐type (black) and transgenic plants (blue). WT: wild‐type. Cyto‐*Sm*NOX: cytosolic *Sm*NOX. Stromal‐*Sm*NOX: chloroplast stromal *Sm*NOX. CIMS‐*Sm*NOX: chloroplast intermembrane space *Sm*NOX. Data represent mean ± 1 SE. (b) As in (a), but for stomatal conductance (*g*_s_, mol/m^2^/s). (c) As in (a), but for leaf temperature (°C). All data represent an average across three independent single copy lines (*n* = 3–5 plants per line). The raw data can be found in Appendix S4.

One important function of the plant transpiration stream is to mediate the temperature control of plant aerial tissues through evaporative cooling. As such, plants exhibiting reduced stomatal numbers might be more susceptible to thermal damage of photosynthesis under supra‐optimal temperatures owing to impairment of their temperature control mechanism. To investigate this, we measured plant photosynthesis across step‐wise increases in air temperatures from 20 to 45 °C. Under these conditions, no difference could be detected in photosynthesis between *Sm*NOX‐expressing lines and wild‐type plants. In all plants, the rate of *A* increased with temperature from 20 °C, reaching an optimum around 30 °C and then rapidly declining with increasing temperatures beyond this optima, consistent with thermal‐induced inactivation of the photosynthetic machinery (Figure 5a). Despite an attenuated rate of *g*_s_ in transgenic plants across all temperature conditions, a comparable trend was also observed in this parameter between both mutant and wild‐type plants over the assay (Figure 5b). Moreover, no difference could be detected in the measured leaf temperature of *Sm*NOX‐expressing lines compared to wild‐type plants at any given air temperature (Figure 5c). Thus, reductions in stomatal density and stomatal conductance in *Sm*NOX‐expressing lines were not associated with either a change in leaf temperature or any change in the susceptibility to thermal damage of the photosynthetic machinery under supra‐optimal temperatures.

### *Sm*NOX improves vegetative growth and biomass accumulation under water‐deficit conditions

**Figure 6:**
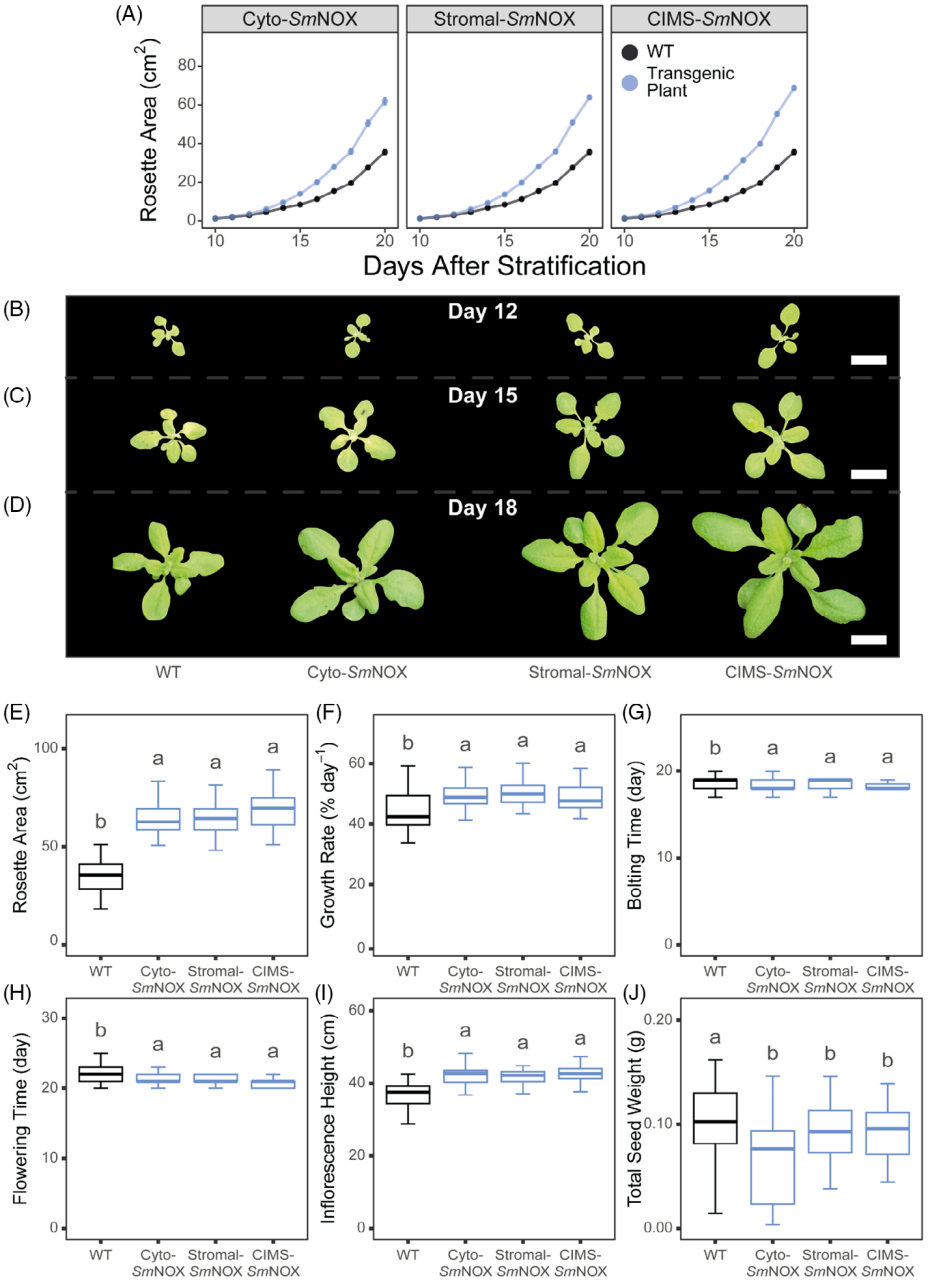
The vegetative and reproductive performance of plants under water‐deficit conditions. (A) The increase in visible rosette area (cm^2^) over time in wild‐type (black) and transgenic plants (blue). WT: wild‐type. Cyto‐*Sm*NOX: cytosolic *Sm*NOX. Stromal‐*Sm*NOX: chloroplast stromal *Sm*NOX. CIMS‐*Sm*NOX: chloroplast intermembrane space *Sm*NOX. Data represent mean ± 1 SE. (B) Representative images of wild‐type and transgenic plants taken on day 12 post stratification. Scale bar = 2.5 cm. (C) As in (B), but for day 15 post stratification. (D) As in (B), but for day 18 post stratification. In parts B–D, the same individual plant is shown across the growing period and all images were generated by computationally removing background from the aerial photographs analysed for growth analyses. (E) Boxplot depicting the maximum visible rosette area at the end of vegetative growth (day = 20). The colour scheme and abbreviations follow that described in (A). (F) As in (E), but for the average percentage growth rate of plants computed over the vegetative growth phase (% increase in rosette area day^−1^). (G) As in (E), but for the time taken for bolting to occur in plants (days post stratification). (H) As in (E), but for the time taken for flowering to occur in plants (days post stratification). (I) As in (E), but for the maximum inflorescence height (cm) measured at day 32 post stratification. (J) As in (E), but for the total seed weight harvested from plants after drying (g). All data represent an average across three independent single copy lines (*n* = 17–20 plants per line for all measurements, except total seed weight where *n* = 11–13 plants per line). Differences between transgenic plants and WT are assessed by Fisher LSD *post‐hoc* analysis following a two‐way ANOVA, where letters above each box represent statistically significant differences in mean values (*P* ≤ 0.05). The raw data can be found in Appendix S4.

Based on the findings that *Sm*NOX expression caused an enhancement in *iWUE* without any off‐target effects on photosynthetic efficiency, stomatal behaviour or thermal sensitivity, we next sought to determine whether an impact on plant growth could be observed. For this purpose, we grew plants in a fully randomized block design under long‐day conditions with a 16‐h photoperiod and 250 μmol/m^2^/s non‐limiting light. Under these conditions, no difference in the vegetative growth or performance between transgenic *Sm*NOX‐expressing plants and wild‐type plants was detected under well‐watered conditions (Appendix S1, Figure S10A–C). Given the higher *iWUE* of mutant plants, we next determined whether these improved photosynthesis‐water relations resulted in any change in plant growth under water‐deficit conditions. For this purpose, plant trays were watered prior to stratification but water was then withheld for 15 days after the termination of stratification to maintain sub‐optimal and deteriorating soil‐water saturation levels during vegetative growth (after which watering was resumed every ~7 days as usual). This assay revealed that *SmNOX‐expressing* plants produced more biomass and larger rosettes compared to wild‐type plants over equivalent periods of time (Figure 6A–E). This result was caused by *Sm*NOX‐expressing lines maintaining higher relative growth rates during water‐deficit (Figure 6F). Consistent with this enhancement in vegetative growth and increased tolerance to adverse water conditions, mutants also exhibited earlier times of bolting (Figure 6G) and flowering (Figure 6H), and produced visibly taller inflorescences relative to wild‐type plants (Figure 6I). However, these alterations in vegetative growth and biomass did not translate to improved yields, and all *Sm*NOX‐expressing lines exhibited a reduction in total seed mass (Figure 6J). Thus, *Sm*NOX‐expressing plants exhibited enhanced biomass production and tolerance to water‐deficit conditions but an overall impairment of seed production.

## Discussion

Manipulation of stomatal density is a key target for enhancing crop productivity and climate resilience under current and future conditions (Bertolino *et al*., 2019; Leakey *et al*., 2019). In the present study, we demonstrate that driving the expression of a water‐forming NAD(P)H oxidase from *Streptococcus mutans* (*Sm*NOX) in stomatal precursor cells causes a reduction in stomatal density in *Arabidopsis thaliana*. We show that this reduced stomatal density phenotype occurs irrespective of whether *Sm*NOX is targeted to the cytosol, chloroplast stroma or chloroplast intermembrane space. We also find that neither *Sm*NOX expression nor *Sm*NOX‐mediated reductions in stomatal density had any detectable impact on plant CO_2_ assimilation rate, the efficiency or thermal sensitivity of photosynthesis, the kinetics of stomatal movement or plant growth under well‐watered conditions. Nonetheless, we discover that all *Sm*NOX‐expressing lines exhibit an enhanced intrinsic water‐use efficiency. We also show that this improvement in water‐use efficiency translates to an increase in plant vegetative growth and biomass accumulation under water‐deficit conditions. Thus, we provide a novel metabolic engineering approach that can be leveraged to alter stomatal patterning and plant photosynthesis‐water relations.

Nicotinamide adenine dinucleotide (NAD(H)) and nicotinamide adenine dinucleotide phosphate (NADP(H)) are ubiquitous coenzymes that facilitate cellular energy transfer, redox homeostasis and signalling in living organisms (Smith *et al*., 2021). Alongside these diverse roles, NAD^+^ has recently been implicated as a negative regulator of stomatal development. Evidence in support of this hypothesis has arisen because perturbations in cellular redox status associated with exogenous NAD^+^ treatment as well as translational repression of either mitochondrial NAD^+^ transporter proteins (*At*NDT1 and *At*NDT2), peroxisomal NAD^+^ transporter proteins (*At*PXN), or poly(ADP‐ribose)polymerase (PARP) proteins involved in NAD^+^ recycling have all produced *A. thaliana* plants with reduced stomatal densities (de Souza Chaves *et al*., 2019; Feitosa‐Araujo *et al*., 2020). The mechanism of this developmental effect is proposed to occur via abscisic acid (ABA) which is known to inhibit entry into the stomatal lineage by suppressing the activity of SPEECHLESS (SPCH) (Tanaka *et al*., 2013), possibly via induction of the cyclin‐dependent kinase (CDK) inhibitor gene ICK1 (Chater *et al*., 2014; Wang *et al*., 1998). This interaction between NAD^+^ and ABA was postulated because NAD^+^ transport and metabolism mutants were associated with both transcriptional changes in ABA metabolic genes and reduced SPCH expression levels (Feitosa‐Araujo *et al*., 2020). Importantly, exogenous NAD^+^ applications were also sufficient to drive a reduction in stomatal patterning in wild‐type *Arabidopsis* but not in mutants impaired in ABA biosynthesis or signalling (Feitosa‐Araujo *et al*., 2020). In the present study, we build on this foundational biology by demonstrating that expression of a water‐forming NAD(P)H oxidase in stomatal precursor cells is capable of driving a reduction in stomatal density through perturbation of endogenous cellular redox status. We further show that such a reduction in stomatal density is achieved irrespective of the subcellular compartment in which this enzyme is targeted. Thus, these results collectively demonstrate that NAD^+^ is a viable metabolic handle that can be turned to alter stomatal patterning in synthetic biology applications.

The density of stomata imposes an upper anatomical constraint on leaf diffusive conductance and thus has important physiological implications for plant productivity (Haworth *et al*., 2011). Despite reduced stomatal density mutants having been generated in a number of disparate species and across many studies (Bergmann *et al*., 2004; Bussis *et al*., 2006; Caine *et al*., 2019; de Souza Chaves *et al*., 2019; Doheny‐Adams *et al*., 2012; Dunn *et al*., 2019; Feitosa‐Araujo *et al*., 2020; Franks *et al*., 2015; Gudesblat *et al*., 2012; Hara *et al*., 2009, 2007; Hepworth *et al*., 2015; Hughes *et al*., 2017; Jalakas *et al*., 2018; Mohammed *et al*., 2019; Pillitteri *et al*., 2007; Wang *et al*., 2016; Yin *et al*., 2017; Yoo *et al*., 2010; Zhao *et al*., 2024), the effects of this developmental alteration on plant photosynthesis, water‐use efficiency, growth and yield traits have proven variable (Lawson and Blatt, 2014). In the present study, we characterize the gas exchange properties of our reduced stomatal density transgenic plants across a range of environmental conditions to shed light on their underlying physiology. We demonstrate that these mutants exhibit an increased intrinsic water‐use efficiency (*iWUE*) by maintaining wild‐type rates of CO_2_ assimilation at lower respective stomatal gaseous conductance. Reductions in stomatal density also caused a tighter coordination between the rates of CO_2_ assimilation and stomatal conductance, such that a reduced extent of water loss occurred after light‐saturated rates of CO_2_ assimilation were achieved. However, decreasing stomatal density did not result in any change in the efficiency or thermal sensitivity of photosynthesis and CO_2_ assimilation, or any change in the kinetics of stomatal movements. Together, these results align with recent findings from EPIDERMAL PATTERNING FACTOR mutants which similarly report reductions in stomatal density without any impact on plant CO_2_ assimilation (Caine *et al*., 2019; Franks *et al*., 2015; Harrison *et al*., 2020; Hughes *et al*., 2017). The biological basis of these collective observations is intriguing. As the rate of photosynthesis at any one time is constrained by the most limiting factor of chloroplast CO_2_ delivery (including both stomatal conductance and mesophyll conductance components) and photosynthetic biochemistry (Gago *et al*., 2019), this phenomenon could be explained if stomata are either not limiting to plant photosynthesis under the study conditions or if secondary adjustments in leaf architecture compensate for changes in stomatal density (Harrison *et al*., 2020). Indeed, the comparable extent of stomatal limitation on photosynthesis measured across wild‐type and *Sm*NOX‐expressing lines herein suggests that some other factor is more limiting than stomatal conductance in these plants. Future research efforts are required to further elucidate this complex interplay between stomata, leaf architecture and plant physiology in *Arabidopsis* as well as in other economically important crop species.

In addition to the lack of effect of reduced stomatal density on leaf photosynthesis, CO_2_ assimilation was also not impacted by the expression of *Sm*NOX under the control of the CAB3 promoter. Thus, whilst *Sm*NOX was able to perturb NAD^+^ levels to mediate a reduction in stomatal density, it was not sufficient to impair photosynthesis. The reason for this is likely because the large mesophyll pool sizes of both NADH (24–55 μm) and NADPH (140–180 μm) (Igamberdiev and Gardeström, 2003) as well as the extraordinarily high rates of mesophyll photosynthetic NADPH production (~70 μmol/g/s) (Wieloch and Sharkey, 2022) would together mask the effect of *Sm*NOX activity in this tissue. By contrast, epidermal cells contribute minimally toward total plant photosynthesis and contain small chloroplasts with a high stroma to grana ratio which would produce much lower rates of NADPH (Barton *et al*., 2018, 2016). Thus, although *Sm*NOX is expressed to a high level throughout diverse photosynthetic tissues when using the CAB3 promoter, the impact of this enzyme on cellular NAD^+^ status would be largest in stomatal progenitor cells.

Consistent with the lack of impairment of stomatal density or *Sm*NOX expression on photosynthetic CO_2_ assimilation, transgenic plants did not exhibit any difference in plant growth or biomass accumulation under well‐watered conditions. However, increased vegetative growth and biomass accumulation were observed under water‐deficit conditions. This improved tolerance to adverse water conditions is caused by the more conservative water input requirements of these plants, as driven by the reduced rates of transpiration and the improvements in photosynthetic water‐use efficiency, and is in agreement with other analyses of reduced stomatal density mutants (Caine *et al*., 2019; Hughes *et al*., 2017; Mohammed *et al*., 2019; Wang *et al*., 2016). Moreover, the reduced leaf mass per area measured in *Sm*NOX‐expressing lines would also contribute toward increased rosette expansion, as requires a lower resource investment per unit leaf tissue produced. Despite this improved tolerance to adverse water conditions, all *Sm*NOX‐expressing lines exhibited a lower overall seed yield. Similar impairments in plant reproductive traits have also been reported in NAD^+^ carrier mutants (de Souza Chaves *et al*., 2019; Feitosa‐Araujo *et al*., 2020) and are likely caused by the role of NAD^+^ in seed (Chai *et al*., 2005; Katoh *et al*., 2006) and pollen development (Cárdenas *et al*., 2006; Hashida *et al*., 2007, 2013). It would be interesting to investigate in future work whether this seed yield impairment is avoidable, and if so whether this could be mitigated by use of other promoters with reduced off‐target expression effects.

## Conclusion

As global food security is increasingly threatened by climate change, developing more climate‐resilient crops presents an important goal for crop breeding and engineering efforts. Altering stomatal density represents a promising avenue to optimize the relationship between plant carbon assimilation, water use, growth and yield. Here, we provide a novel metabolic engineering strategy which can be exploited to reduce plant stomatal density by altering the endogenous NAD^+^ status of stomatal precursor cells. We demonstrate that this approach produces plants with improved photosynthesis‐water relations and improved productivity under water‐deficit conditions without any apparent cost on photosynthetic carbon assimilation or growth under normal conditions. Thus, we reveal a novel avenue by which plants may be altered to enhance productivity and climate resilience.

## Materials and methods

### Gene accession numbers

The following list comprises the accession numbers of the genes used in this study: *Sm*NOX2 (WP_002268749.1); *At*RBCS1A (At1g67090); *At*PSBQA (At4g21280); *At*OEP9 (At1g16000).

### Gene synthesis

A variant of the NAD(P)H oxidase (NOX) enzyme encoded by the Streptococcus mutans *nox2* gene but containing amino acid substitutions D192A/V193R/V194H/A199R was chosen for use in this study (Petschacher *et al*., 2014). This gene (termed *Sm*NOX) was codon optimized for expression in *Arabidopsis thaliana* and domesticated to remove recognition sites for the type IIS restriction enzymes BsaI or BpiI. Appropriate flanking regions compatible with Golden Gate Assembly (Marillonnet and Engler, 2014) were added to the 5' and 3' regions, and the resulting coding sequence was commercially synthesized (TWIST Bioscience, South San Francisco, CA, USA). The full‐length coding sequence of *At*OEP9 and *At*RBCS1A, as well as a chloroplast stromal transit peptide from *At*PSBQA (containing the first 87 amino acids of *AtPSBQA* including the intact stromal import domain but lacking a complete thylakoid targeting motif (Ma *et al*., 2021)) was subject to these same steps for codon optimisation, domestication, addition of compatible flanking regions for Golden Gate assembly (Marillonnet and Engler, 2014) and gene synthesis. The full‐length sequences of each of these genes are found in Appendix S2.

### Construct design and assembly

Genetic constructs for stable expression in plants were generated by cloning the coding sequence of *Sm*NOX downstream of the *A. thaliana* chlorophyll *a*/*b*‐binding protein 3 promoter (*At*CAB3; pTEI071) and upstream of the *A. tumefaciens* nopaline synthase terminator (nosT; piCH41421) in the Level 1 backbone position forward 2 vector pICH47742. Compositional forms of this Level 1 module were also generated in which *Sm*NOX was translationally fused at the N‐terminus to either the *At*PSBQA transit peptide or the *At*OEP9 full‐length coding sequence, respectively. Each of these constructs were subsequently assembled into Level 2 Golden Gate modules using the plant transformation vector acceptor backbone pAGM37443 (Grützner *et al*., 2021) with the pFAST‐R seed‐coat specific fluorescent selection cassette (Engler *et al*., 2014) (pICSL70008) and the end Linker position 3 insert (pICH49266).

Genetic constructs for transient expression in protoplasts were generated by cloning the coding sequence of *Sm*NOX translationally fused to a C‐terminal enhanced green fluorescent protein (GFP; pJOG176) downstream of the cauliflower mosaic virus 35S promoter (CaMV35S; EC15058) and upstream of the *A. tumefaciens* nosT (piCH41421) in the Level 1 backbone position forward 2 vector pICH47742. As above, compositional forms of this construct were also generated in which *Sm*NOX was translationally fused at the N‐terminus to the *At*PSBQA transit peptide or the *At*OEP9 full‐length coding sequence, respectively. Positive control constructs were also cloned using the same promoter, terminator and backbone vector but containing only the coding sequence of the GFP (cytosolic fluorescence control) or the coding sequence of GFP translationally fused at the N‐terminus to the full‐length *At*RBCS1A gene sequence (chloroplast stromal fluorescence control). All cloning reactions were performed following the Golden Gate one‐step one‐pot protocol (Engler *et al*., 2009) as described in Appendix S1. The provenance of commercial vectors used during cloning are also provided in Appendix S1. Full‐length sequences of each expression cassette generated are provided in Appendix S2. The full set of oligonucleotides used for cloning, colony PCR and Sanger sequencing are found in Appendix S3. The thermocycler conditions used for all PCR reactions are also described in Appendix S3.

### Protoplast isolation, transformation and visualization

Mesophyll protoplasts were isolated from leaves of 4‐week‐old wild‐type *Arabidopsis thaliana* Col‐0 plants following the ‘Tape‐*Arabidopsis* Sandwich’ method (Wu *et al*., 2009). Protoplast transformation was performed using the calcium/polyethylene glycol‐mediated method (Yoo *et al*., 2007), as described in (Wu *et al*., 2009). Transformed protoplasts were incubated in the dark overnight at 25 °C prior to imaging. Cell fluorescence was visualized using a Zeiss LSM 880 Airy Scan confocal microscope (Zeiss, Oberkochen, Germany). Zeiss ZEN software (Zeiss) was used for confocal laser scanning microscopy and subsequent image processing.

### Plant material and stable transformation

Stable transformation of *A. thaliana* ecotype Columbia (Col‐0) background was performed using the agrobacterium‐mediated floral dip method (Clough and Bent, 1998). Positive transformants were initially screened based upon seed fluorescence visualized by epifluorescence microscopy (Leica M165 stereo microscope, Leica Microsystems, Wetzlar, Germany) and confirmed by genotyping PCR and gel electrophoresis using the OneTaq® 2X Master Mix with Standard Buffer (New England Biolabs, Catalogue #M0482S, Ipswich, MA, USA) following the manufacturer's instructions for reaction set‐up and the oligonucleotides and thermocycler conditions described in Appendix S3. Single insertion events were subsequently identified by TaqMan® real‐time PCR with fluorescent probes and primers which bind to the TagRFP selectable marker (iDNA GENETICS, Norwich, UK), and homozygous individuals were isolated. For the above purposes of genotyping PCR and TaqMan® real‐time PCR, genomic DNA was isolated from mature leaves of T1 plants using the cetyl trimethylammonium bromide (CTAB) method (Murray and Thompson, 1980). In total, three single‐copy homozygous lines were taken forward as the basis of the analysis herein for each *Sm*NOX‐expressing transgenic plant (cyto‐*Sm*NOX, stromal‐*Sm*NOX and CIMS‐*Sm*NOX). All experiments described are performed on T3 and T4 homozygous plants.

### Stomatal density measurements

Epidermal impressions from the abaxial surface of healthy and fully expanded excised leaves of 8‐week‐old plants were obtained following the clear nail polish method (Wu and Zhao, 2017). All leaves used for this analysis were of similar size. After mounting on microscope slides, stomatal density was measured using a Leica DMR8 light microscope (Leica Microsystems, Wetzlar, Germany) at ×20 magnification under bright‐field view. Stomatal density counts were calculated across five independent fields of view of the same leaf, and across a total of 9–10 biological replicates per line.

### Leaf mass area measurements

A leaf puncher was used to harvest a fixed area of tissue from healthy and fully expanded leaves of 8‐week‐old plants. Leaf dry mass was subsequently determined after 48 h incubation at 65 °C. All leaves were of similar size and were harvested in the middle of the photoperiod. Leaf mass area (LMA, g/m^2^) was subsequently determined as the fixed leaf area divided by leaf dry mass.

### *In vitro* enzyme assays

A consistent area of leaf tissue was harvested from healthy and fully expanded leaves of 6‐week‐old plants using a leaf puncher during the middle of the photoperiod. Leaf material was flash frozen in liquid nitrogen, ground using metal beads and a tissue lyser (TissueLyser II, QIAGEN, Hilden, Germany) and resuspended in 500 μL ice‐cold extraction buffer (50 mm potassium phosphate, 5 mm MgCl2, 1 mm EDTA, 3% Triton X‐100) with 1x protease inhibitor cocktail (Sigma‐Aldrich, Catalogue #P9599‐5ML, St. Louis, MI, USA). Homogenate was centrifuged for 1 min at 17 800 ***g*** at 4 °C, and total crude protein extract was isolated from the supernatant.

Enzyme assays were performed following the method described in (Petschacher *et al*., 2014) with some alterations. In brief, all reactions included a total volume of 200 μL in unsealed 96 well plate at 25 °C. Typical reaction mixtures contained 50 mm potassium phosphate buffer (pH 7.5) with 250 μm of either NADH or NADPH. Reactions were vortexed for saturation with oxygen in air (250 μm), and were initiated by the addition of 30 μL undiluted crude leaf lysate. Negative controls containing ddH_2_O in place of leaf lysate were included in every assay. Consumption of NADH or NADPH was monitored spectrophotometrically at 340 nm using a plate reader (FLUOstar Omega, BMG Labtech, Ortenberg, Germany). Each biological replicate was repeated over two technical replicate wells, and rates of enzyme reaction were calculated over the linear part of the curve over a duration of at least 5 min. Reaction rates were normalized per unit NADH/NADPH oxidation by standard curve.

### Leaf gas exchange

Gas exchange analyses were performed using a LI‐6800 open‐path gas exchange system equipped with a 6800‐01A fluorometer head (LI‐COR, Lincoln, NE, USA) using the 2 cm^2^ leaf chamber and 90% red and 10% blue actinic light under normal 21% O_2_ air. The youngest fully expanded leaf was used for each plant and all measurements were taken prior to onset of flowering in the developmental window between principal rosette growth stage 3.50 and 3.70, according to (Boyes *et al*., 2001). Downstream statistical analysis, model fitting and parameter estimation from leaf gas exchange data was performed as described in Appendix S1.

Response of plant gas exchange to light followed the method of (López‐Calcagno *et al*., 2020) with some alterations. In brief, plants were first adapted at 2200 μmol/m^2^/s for 20 min, and photosynthesis was then measured at step‐wise intervals of 2200, 2000, 1650, 1300, 1000, 750, 500, 400, 300, 200, 150, 100, 50, 0 and 2200 μmol/m^2^/s light. The environment was held constant at 400 μmol/mol CO_2_, 30 °C leaf temperature, 55% relative air humidity, 500 μmol/s flow rate and 10 000 rpm fan speed. No matching was performed during the analysis, and a 4‐min wait time was used between each light condition.

For the response of plant gas exchange to intercellular CO_2_, plants were first adapted at 400 μmol/mol CO_2_ for 20 min, and photosynthesis was then measured at step‐wise intervals of 400, 0, 50, 75, 100, 200, 400, 800, 1200 and 1600 μmol/mol reference atmospheric CO_2_ concentrations. The environment was held constant at 1500 μmol/m^2^/s light, 30 °C leaf temperature, 55% relative air humidity, 500 μmol/s flow rate and 10 000 rpm fan speed. Matching of CO_2_ and H_2_O was performed before each measurement, with a minimum and maximum wait time of 3 and 4 min, respectively.

Investigation of stomatal kinetics in response to step changes in light followed the method of (McAusland *et al*., 2016). In brief, plants were first adapted to 100 μmol/m^2^/s light for 60 min and were then subjected to a step increase to 1000 μmol/m^2^/s for 60 min, followed by a step decrease back to 100 μmol/m^2^/s for 30 min. The environment was held constant at 400 μmol/mol CO_2_, 30 °C leaf temperature, 1.8 kPa vapour pressure deficit, 500 μmol/s flow rate and 10 000 rpm fan speed. No matching was performed during the analysis, and measurements were taken every minute.

For the response of plant gas exchange to temperature, plants were first adapted at 20 °C for 20 min and photosynthesis was then measured at step‐wise intervals of 20, 25, 30, 35, 40 and 45 °C reference air temperatures. The environment was held constant at 1500 μmol/m^2^/s light, 400 μmol/mol CO_2_, 500 μmol/s flow rate and 10 000 rpm fan speed. As previously described in the thermal analysis of (Busch and Sage, 2017), it is difficult to maintain VPD at temperatures above 30 °C. Thus, leaf vapour pressure deficit (VPD) was maintained at 1.5 kPa for measurements at 20, 25 and 30 °C, 2.5 kPa for the measurement at 35 °C, 3.5 kPa for the measurement at 40 °C and 4.5 kPa for the measurement at 45 °C. Matching of CO_2_ and H_2_O was performed before each measurement with a minimum and maximum wait time of 10 and 14 min, respectively.

### Plant growth conditions

Seeds were grown in individual pots directly on soil (Levington Seed modular compost) and were stratified for 2 days at 4 °C to overcome dormancy. Unless explicitly stated, plants were grown in climate‐controlled environmental conditions in floor‐standing growth chambers (FitoclimaD1200 PLH, Aralab, Sintra, Portugal) under short‐day conditions (8‐h light/16‐h dark cycle) at 21 °C, ambient 400 ppm CO_2_, 50% relative humidity and 125 μmol/m^2^/s light and were maintained under well‐watered conditions.

### Plant growth assays

For analysis of plant growth, stratified seeds were transferred to controlled environment chambers (CERs) at The University of Oxford and grown under a 16‐h/8‐h day/night period, 21 °C and 250 μmol/m^2^/s light. To minimize confounding and positional effects, plant genotypes were grown in a fully randomized block design and the position of trays were rotated every 2 days. For well‐watered experiments, plants were watered on a weekly basis as usual from the base of the tray. For the water‐deficit growth experiment, plant trays were watered prior to stratification as above, but water was then withheld completely for 15 days after germination to maintain sub‐optimal and deteriorating soil‐water saturation levels during vegetative growth. After this water‐deficit period of 15 days, watering was resumed as described in the well‐watered growth experiments.

Aerial photographs were taken daily during vegetative growth. Image processing was performed to correct for orientation and perspective using Yet Another Scanning Wizard (YASW) (https://github.com/ImageProcessing‐ElectronicPublications/yasw). Computational analysis was then performed to derive rosette growth parameters. Green pixels were isolated from images using a purpose‐built script, and visible rosette area was computed with ImageJ software (Schneider *et al*., 2012) using the in‐built ‘Analyse Particles’ function. Time of bolting was measured as the number of days after stratification when the inflorescence was greater than 1 cm in height above the rosette. Time of flowering time was measured as the number of days after stratification at which the first flower was open and petals were visible. Plant height was measured as the maximum height of the primary inflorescence from the base of the rosette at 32 days post stratification. Total seed weight was measured after allowing plants to completely dry for at least a month.

## Conflict of interest

SK is co‐founder of Wild Bioscience Ltd. SK and JWB have applied for a patent on the work presented in this study.

## Author contributions

JWB and SK conceived the study, designed the experiments, interpreted and analyzed the data and wrote the manuscript. JWB conducted the experiments.

## Funding

This work was funded by the Royal Society and the European Union's Horizon 2020 research and innovation program under grant agreement number 637765. JWB was funded by the BBSRC through BB/J014427/1. This research was funded in whole or in part by the BBSRC number BB/J014427/1. For the purpose of open access, the author has applied a CC BY public copyright licence to any Author Accepted Manuscript version arising from this submission.

## Supporting information


**Appendix S1** Supplemental results, materials and methods, figures, and tables.

**Appendix S2** List of nucleotide and protein sequences.

**Appendix S3** List of oligonucleotide sequences and thermocycler conditions.

**Appendix S4** Raw data supporting figures and tables.

**Appendix S5** Processed transcriptome datasets.

## Data Availability

All data used in this study is provided in the supplemental material.
