# Supplementary material for: Metabolic engineering of stomatal precursor cells enhances photosynthetic water‐use efficiency and vegetative growth under water‐deficit conditions in Arabidopsis thaliana
Source: Plant Biotechnol J. 2025 May 23;23(8):3177–94. doi: 10.1111/pbi.70130 (PMC12310843; doi:10.1111/pbi.70130)
Supplement: Supplementary file 1 — Appendix S1 Supplemental results, materials and methods, figures, and tables. [file PBI-23-3177-s004.docx]

# Supplemental Results

## NOX expression results in tighter coordination between rates of assimilation and stomatal conductance

To further investigate the *iWUE* phenotype, the relative coordination between *A* and *g*_s_ was evaluated across different light intensities in the light-response curve. For this purpose, values of *g*_s_ were compared between *g*_s sat_ and that observed at the modelled light intensity where 95% maximum assimilation was achieved (*g_s_ _A95_*), beyond which neither CO_2_ delivery nor light is considered limiting to photosynthesis. This revealed that rates of *g*_s_ in wild-type plants continue to increase with light by 8.1% after *g_s_ _A95_* was attained, without resulting in any benefit in terms of CO_2_ assimilation (Figure 3G, Supplemental File 1, Figure S6A – S6C, table S3). In contrast, the magnitude of this “overshooting” behaviour was significantly dampened in transgenic plants, where an increase in *g_s_* of only 3.3 – 4.4% was observed with further increases in light after *g_s_ _A95_* was attained (Figure 3G, Supplemental File 1, Figure S6A – S6C, table S3). This same result was recapitulated when comparing the respective light intensities at which 95% maximum assimilation rate (PPFD _A95_) and *g*_s sat_ (PPFD *g*_s sat_) were achieved. For instance, values of PPFD _A95_ and PPFD *g*_s sat_ were more similar to one another in transgenic (cyto-*Sm*NOX: 828.6 ± 161.3 μmol m^-2^ s^-1^, 156.2%; stromal-*Sm*NOX: 621.4 ± 142.8 μmol m^-2^ s^-1^, 124.6%; CIMS-*Sm*NOX: 847.6 ± 169.7 μmol m^-2^ s^-1^, 168.2%) compared to wild-type plants (1385.7 ± 235.5 μmol m^-2^ s^-1^, 290.0%) (Figure 3H, Supplemental File 1, Figure S6D – S6F, table S3). Thus, the reduced stomatal density caused by *Sm*NOX expression resulted in an enhanced water-use efficiency and tighter coupling of *g*_s_ and *A* across different light conditions.

# Supplemental Materials and Methods

## Cloning and construct assembly

Cloning was performed using the Golden Gate one-step one-pot protocol ^1^ with modified thermocycler conditions consisting of 80 cycles of 5 minute digestion (37°C) and 5 minute ligation (16°C) steps, respectively. All reactions contained T4 DNA ligase (New England Biolabs, Catalog #M0202S) and the Type IIS restriction enzymes BsaI-HF®v2 (Level 1 assembly; New England Biolabs, Catalog #R3733S) or BpiI (Level 2 assembly; Thermo Fisher Scientific, Catalog # ER1012). Assembled plasmids were checked by colony PCR and gel electrophoresis using the OneTaq® 2X Master Mix with Standard Buffer (New England Biolabs, Catalog #M0482S) using generic primers that bind to vector backbones, and sequences of inserts were confirmed by Sanger sequencing (Source BioScience, Nottingham, United Kingdom). Full plasmid sequencing (Plasmidsaurus, South San Francisco, California, United States) was performed on all Level 2 constructs prior to transformation. Where 5’ and 3’ overhangs of vectors were not compatible for a given reaction, gene inserts were amplified by PCR with modified overhangs and resulting linear fragments were used directly for cloning. All PCR reactions for cloning were performed using Q5® High-Fidelity DNA Polymerase (New England Biolabs, Catalog #M0491S) following manufacturer’s instructions for reaction set-up.

## Vectors

pTEI071 and pJOG176 were a gift from Johannes Stuttmann (Addgene plasmid #105376; http://n2t.net/addgene:105376; RRID:Addgene_105376, and Addgene plasmid #105392; http://n2t.net/addgene:105392; RRID:Addgene_105392, respectively) ^2^**.** piCH41421 was a gift from Sylvestre Marillonnet and Nicola Patron (Addgene plasmid #50339; http://n2t.net/addgene:50339; RRID:Addgene_50339) ^3^. pICSL70008 was a gift from Nicola Patron (Addgene plasmid #50336; http://n2t.net/addgene:50336; RRID:Addgene_50336) ^3^. pICH47742 and pICH49266 were a gift from Sylvestre Marillonnet (Addgene plasmid # 48001; http://n2t.net/addgene:48001; RRID:Addgene_48001, and Addgene plasmid #48024 ;http://n2t.net/addgene:48024; RRID:Addgene_48024) ^4^**.** pAGM37443 was a gift from Sylvestre Marillonnet (Addgene plasmid #153218; http://n2t.net/addgene:153218; RRID:Addgene_153218) ^5^. EC15058 was a gift from Ben Miller.

## Statistical analysis, model fitting, and parameter estimation of leaf gas exchange data

For statistical analysis of the response of plant gas exchange to light, non-linear curves were fit to the data by implementing the non-rectangular hyperbola model ^6^ using the ‘fit_photosynthesis’ function in the R package photosynthesis V2.1.4 ^7,8^. Values of light compensation point, mitochondrial respiration in the light, apparent quantum yield, and light-saturated electron transport rate were derived from these fitted models. Light-saturated rates of assimilation (*A*_sat_), stomatal conductance, and intrinsic water-use efficiency were manually determined as the highest value of these respective traits across the light response curve. The 95% maximum assimilation rate was also computed manually from the fitted models.

For statistical analysis of the response of plant gas exchange to CO_2_, the Farquhar- von Caemmerer- Berry model of leaf photosynthesis was fit to the data using the ‘fitaci’ function from the R package plantecophys ^9^. For this purpose, the bilinear method was used with triose phosphate utilization implemented to improve model fitting. Values of the maximum carboxylation rate of rubisco (*V*_cmax_), maximum electron transport rate (*J*_max_), CO_2_ compensation point, the Michaelis constant of rubisco for CO_2_ in 21% O_2_ air, and the CO_2_ concentration at the transition between rubisco-limited and ribulose-1,5-bisphosphate (RuBP) regeneration-limited photosynthesis were all derived from these fitted models using this function. Estimates of *V*_cmax_ and *J*_max_ re-scaled to 25°C were also generated after in-built temperature corrections of the data using the ‘fitaci’ function ^9^. The CO_2_–saturated assimilation rate (*A*_max_) was manually determined as the highest value of this trait across the CO_2_ response curve. The CO_2_ concentration at the transition between RuBP regeneration-limited photosynthesis and triose-phosphate utilization-limited photosynthesis were not reported as could not be estimated in all cases. Stomatal limitation (*L*_s_) was computed from the response of plant gas exchange to CO_2_ using the formula [*L*_s_ = (*A*_0_ – *A*) / *A*_0_] following the method of ^10^. Where, *A* is equal to the CO_2_ assimilation rate measured at the initial ambient reference [CO_2_] of 400 µmol mol^-1^, and *A*_0_ is equal to the estimated CO_2_ assimilation rate which would occur under ambient conditions if resistance to CO_2_ diffusion was zero (i.e., when *C*_i_ is equal to 400 μmol mol^-1^ CO_2_).

Statistical analysis of stomatal kinetic parameters to step changes in light intensity between 100 μmol m^−2^ s^−1^ and 1000 μmol m^−2^ s^−1^ were derived using a dynamic sigmoidal mathematical model ^11,12^. For this purpose, model fitting was implemented using the licornetics V2.1.2 source code downloaded from the github repository <https://github.com/lbmountain/licornetics>. Estimates of the maximal rate of stomatal opening, time taken to achieve new steady-state stomatal conductance, and the initial lag in response time of *g*_s_ to the step increase in light intensity from 100 μmol m^−2^ s^−1^ to 1000 μmol m^−2^ s^−1^, as well as the estimated time taken to achieve new steady-state stomatal conductance after the step decrease in light intensity from 1000 μmol m^−2^ s^−1^ to 100 μmol m^−2^ s^−1^ were all derived from models fitted in this way. Prior to interrogation of stomatal kinetic parameters, automated outlier detection based on *A* and *g*_s_ raw data obtained during the initial 100 μmol m^-2^ s^-1^ light phase was performed using the Tukey method so as to remove biological replicates showing high instability.

# Supplemental Figures

## Supplemental File 1, Figure S1


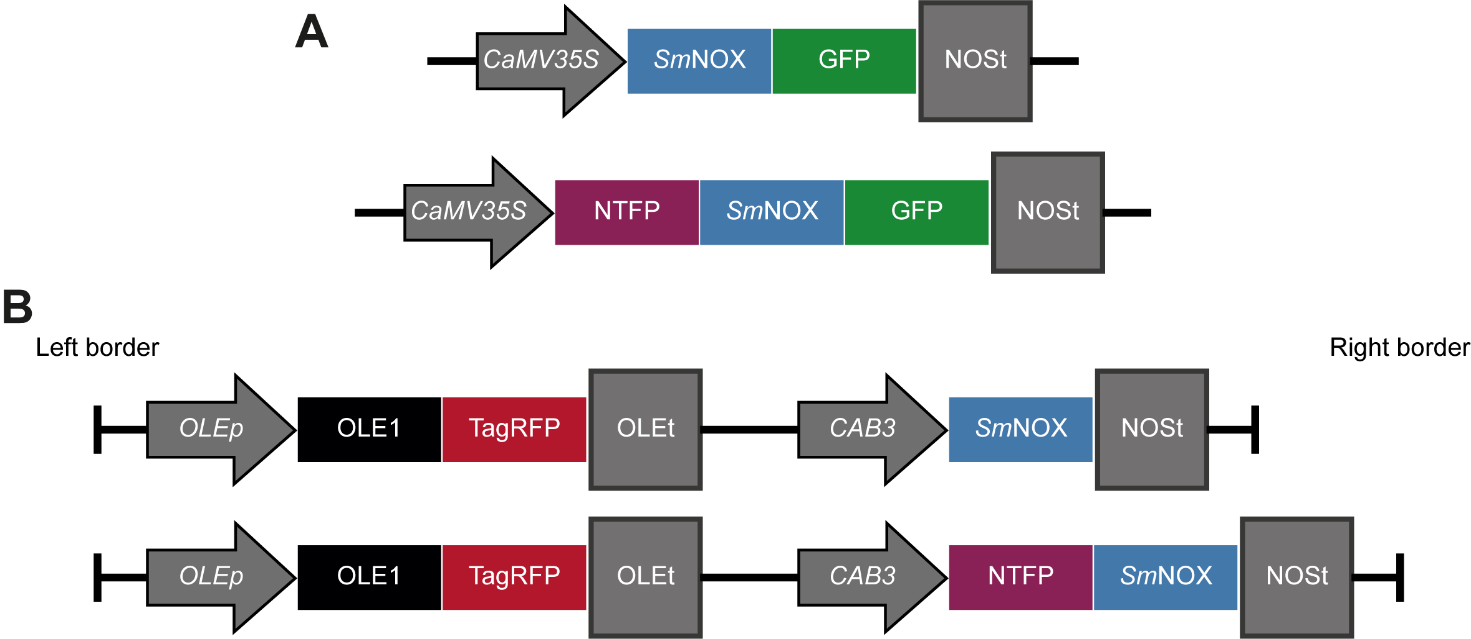


**Figure S1.** *Sm*NOX expression cassettes used for experiments in *Arabidopsis thaliana*. A) Simplified schematic of the expression cassette used for *Sm*NOX transient expression in *A. thaliana* protoplasts. The construct contains a CaMV 35S promoter (CaMV35S) and a nopaline synthase terminator (NOSt) controlling the expression of the *Sm*NOX coding sequence translationally fused to eGFP at the C-terminus. Top: untargeted *Sm*NOX. Bottom: *Sm*NOX targeted by an N-terminal fusion peptide (NTFP). NTFPs are derived from *A. thaliana* genes and include the predicted chloroplast stromal transit peptide from the photosystem II subunit QA gene or the full-length outer envelope protein 9 gene. B) As in (A), but a simplified schematic of the expression cassette used for *Sm*NOX stable expression in *A. thaliana*. In contrast to (A), *Sm*NOX does not contain a C-terminal eGFP and is driven under the control of the chlorophyll a/b-binding protein 3 (CAB3) promoter. All constructs also include an additional expression cassette encoding a seed coat specific fluorescent selectable marker which consists of monomeric TagRFP from *Entacmaea quadricolor* fused to the coding sequence of the *Arabidopsis thaliana* oleosin1 gene (OLE1) under the control of the native oleosin1 promoter (OLEp) and terminator (OLEt), respectively. The complete nucleotide and amino acid sequences for each expression cassette can be found in Supplemental File 2.

## Supplemental File 1, Figure S2


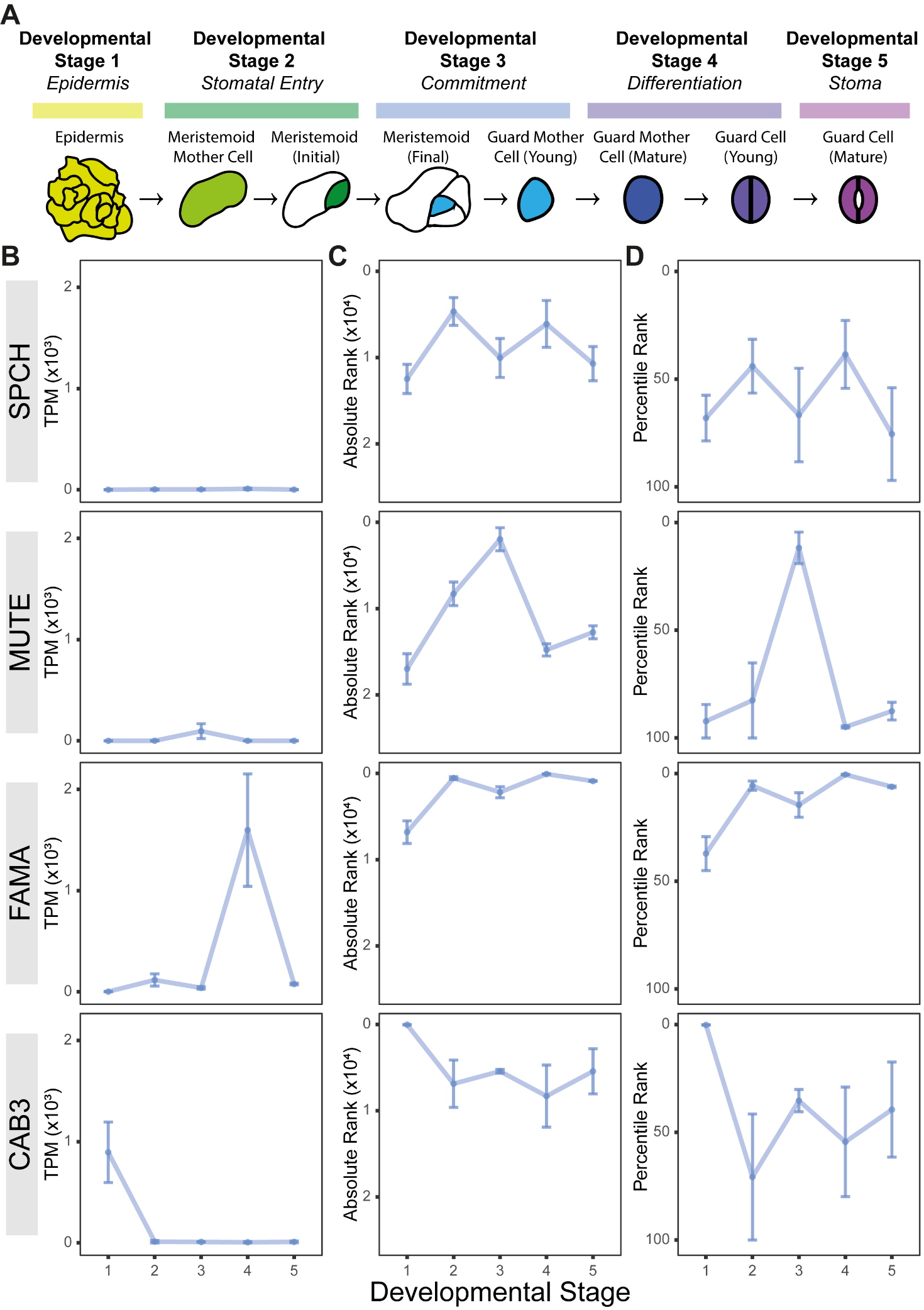


**Figure S2.** The chlorophyll a/b binding protein (CAB3) promoter drives high gene expression in stomatal precursor cells. A) Schematic of the developmental trajectory of stomatal lineage differentiation including all major steps and fate transitions from undifferentiated epidermal cell (Developmental Stage 1), stomatal lineage entry (Developmental Stage 2), stomatal commitment (Developmental Stage 3), stomatal differentiation (Developmental Stage 4), and finally, development of a fully-functional mature stoma (Developmental Stage 5). Figure adapted from ^13^. B) Temporal profile of transcript abundance (TPM; Transcripts Per Million) along the stomatal developmental trajectory in leaves of *Arabidopsis thaliana* for SPEECHLESS (SPCH), MUTE, FAMA, and CAB3 genes, respectively. Developmental stages follow those described in (A). Representative transcriptomes of populations of cells at each developmental stage were obtained from ^13^. C) As in (B), but for the transcript abundance of SPEECHLESS (SPCH), MUTE, FAMA, and CAB3 genes expressed as a rank against the distribution of the transcript abundances of all other nuclear-encoded genes in *A. thaliana*. Ranks are calculated in descending order of gene transcript levels (i.e., low ranks denote genes with the highest relative expression and *vice versa*) and the y-axis has been inverted for ease of visualisation. D) As in (B), but for the transcript abundance of SPEECHLESS (SPCH), MUTE, FAMA, and CAB3 genes expressed as a percentile rank against the distribution of the transcript abundances of all other nuclear-encoded genes in *A. thaliana*. Gene percentile ranks are calculated in descending order of gene transcript levels (i.e., low percentile ranks denote genes with the highest relative expression and *vice versa*) and the y-axis has been inverted for ease of visualisation. The raw data can be found in Supplemental File 5.

## Supplemental File 1, Figure S3


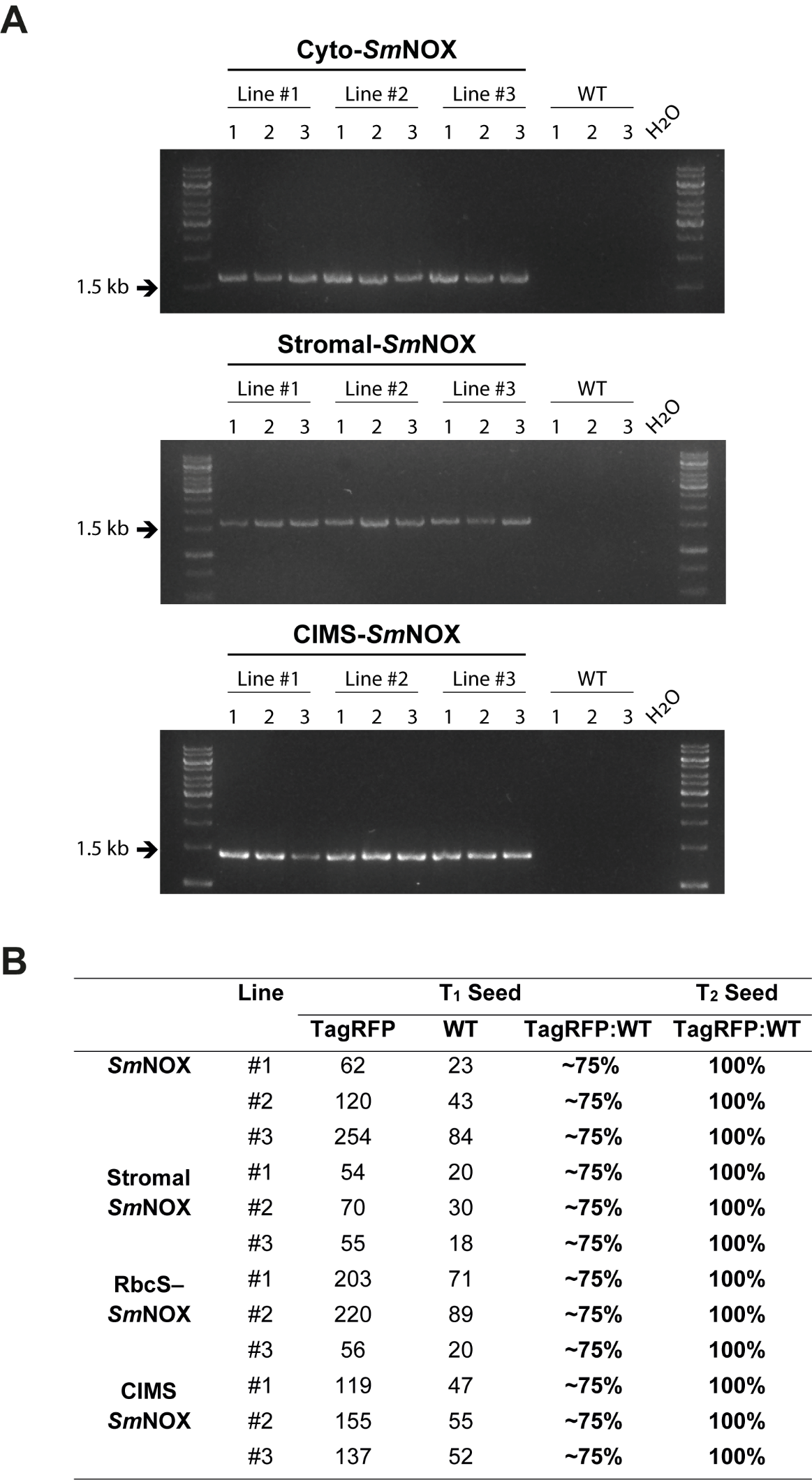


**Figure S3.** Verification of *Sm*NOX implementation. A) Genotyping of transgenic T_3_ *Sm*NOX and wild-type (WT) plants based on primers specific for each gene insertion. The primers, reaction set up, and thermocycler conditions used for polymerase chain reaction (PCR) can be found in Supplemental File 3. B) Segregation analysis data of seed from T_1_ and T_2_ generation plants.

## Supplemental File 1, Figure S4


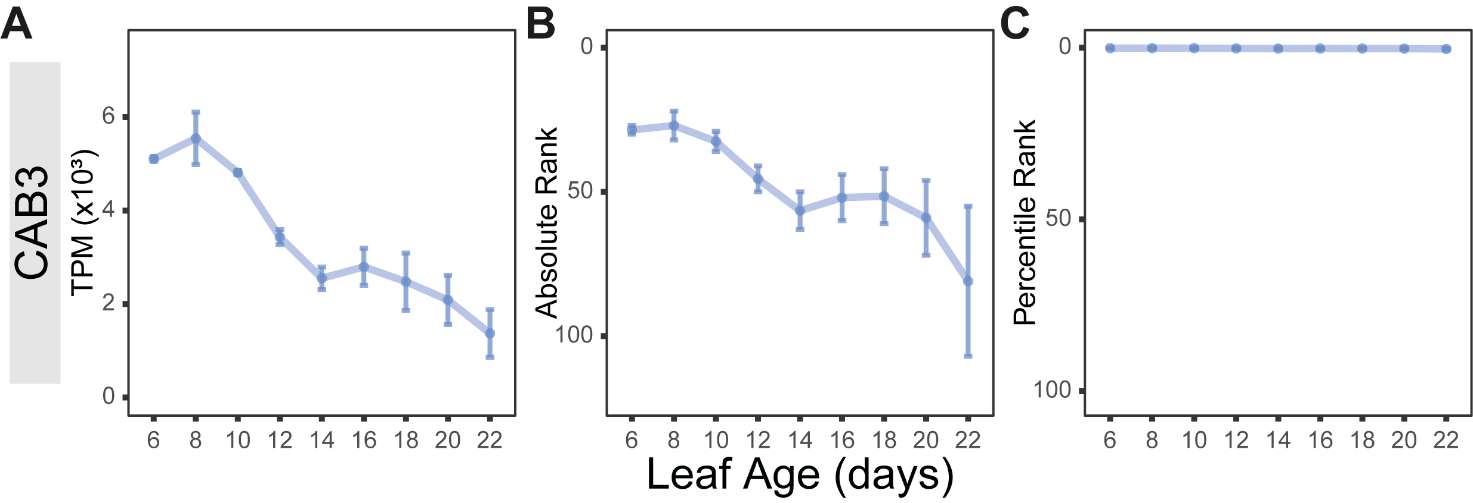


**Figure S4.** The chlorophyll a/b binding protein (CAB3) promoter drives high levels of gene expression in leaves. A) Temporal profile of CAB3 transcript abundance (TPM; Transcripts Per Million) along a gradient of leaf aging in *Arabidopsis thaliana*. Representative transcriptomes from leaves at each age were obtained from ^14^. B) As in (A), but for the transcript abundance of the CAB3 gene expressed as a rank against the distribution of transcript abundances of all other nuclear-encoded genes in *A. thaliana*. Gene ranks are calculated in descending order of gene transcript levels (i.e., low ranks denote genes with the highest relative expression and *vice versa*) and the y-axis has been inverted for ease of visualisation. C) As in (A), but for the transcript abundance of the CAB3 gene expressed as a percentile rank against the distribution of all other nuclear-encoded genes in *A. thaliana*. Gene percentile ranks are calculated in descending order of gene transcript levels (i.e., low percentile ranks denote genes with the highest relative expression and *vice versa*) and the y-axis has been inverted for ease of visualisation. The raw data can be found in Supplemental File 5.

## Supplemental File 1, Figure S5


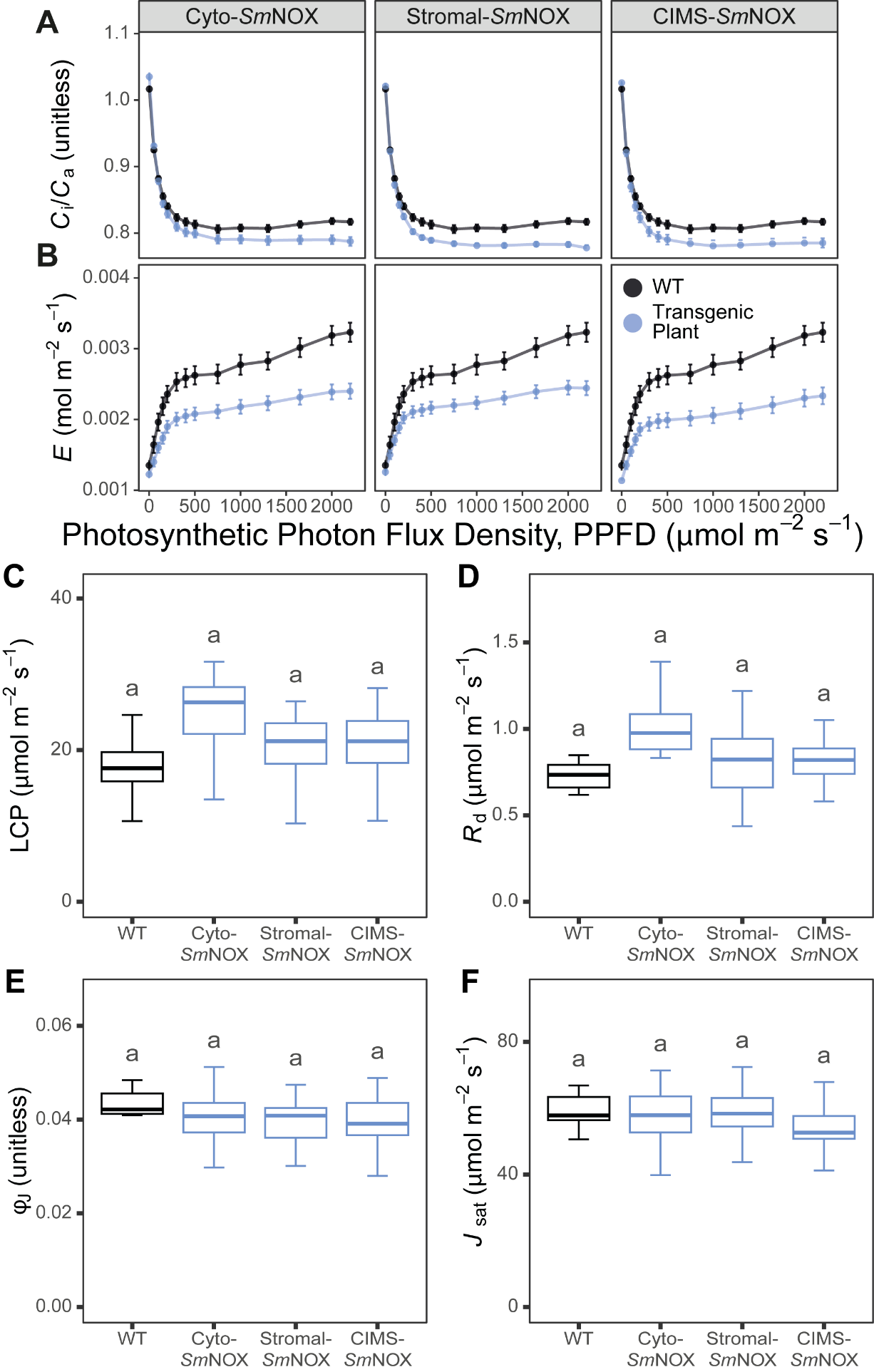


**Figure S5.** Additional parameters computed from the response of plant gas exchange to light (photosynthetic photon flux density: PPFD, μmol m^-2^ s^-1^). A) The ratio of intercellular CO_2_ to ambient CO_2_ (*C*_i_/*C*_a_, unitless) at different PPFD in wild-type (black) and transgenic plants (blue). WT: wild-type. Cyto-*Sm*NOX: cytosolic *Sm*NOX. Stromal-*Sm*NOX: chloroplast stromal *Sm*NOX. CIMS-*Sm*NOX: chloroplast intermembrane space *Sm*NOX. Data represent mean ± 1 S.E. B) as in (A), but for plant transpiration rate (*E*, mol m^−2^ s^−1^). C) Boxplot depicting the light compensation point (LCP, μmol mol^−1^). The colour scheme and abbreviations follow that described in (A). D) As in (C), but for the mitochondrial respiration in the light (*R*_d_, μmol m^−2^ s^−1^). E) As in (C), but for the modelled apparent quantum yield (ΦJ, μmol m^−2^ s^−1^). F) As in (C), but for the light-saturated electron transport rate (*J*sat, μmol m^−2^ s^−1^). All data represent an average across three independent single copy lines (*n* = 7 – 8 per individual line). Differences between transgenic plants and WT are assessed by Fisher LSD post-hoc analysis following a two-way ANOVA, where letters above each box represent statistically significant differences in mean values (p ≤ 0.05). The raw data can be found in Supplemental File 4.

## Supplemental File 1, Figure S6


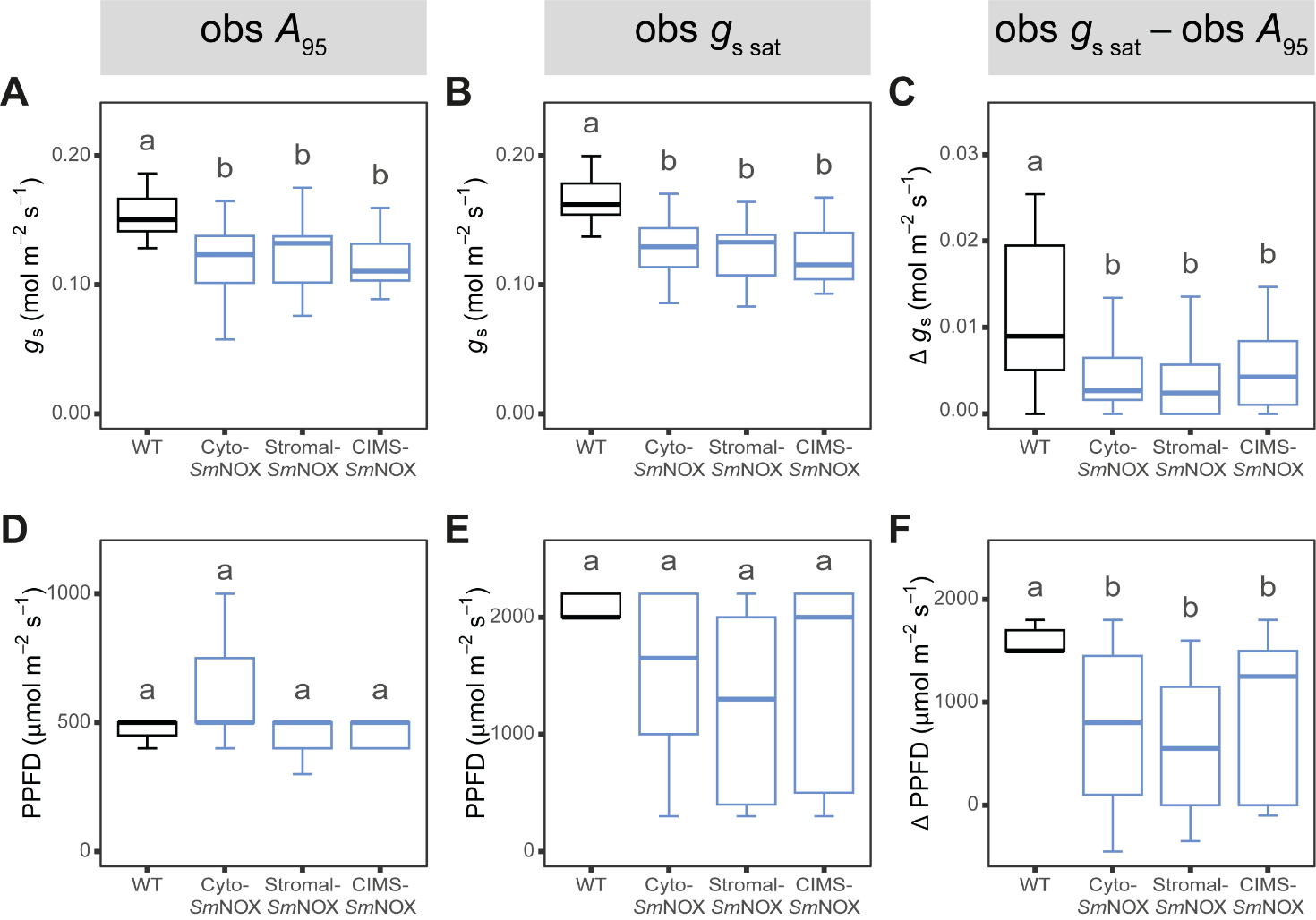


**Figure S6.** The relative coordination between rates of plant photosynthesis and gas exchange across light response curves. A) Boxplot depicting the stomatal conductance at 95% maximum assimilation rate (*g_s_ _A95_*, mol m^−2^ s^−1^) in wild-type (black) and transgenic plants (blue). WT: wild-type. Cyto-*Sm*NOX: cytosolic *Sm*NOX. Stromal-*Sm*NOX: chloroplast stromal *Sm*NOX. CIMS-*Sm*NOX: chloroplast intermembrane space *Sm*NOX. B) As in (A), but for the light-saturated stomatal conductance (*g*_s sat_, mol m^−2^ s^−1^). C) As in (A), but for difference in stomatal conductance at 95% maximum assimilation rate and the light-saturated stomatal conductance rate (Δ *g*_s_, mol m^-2^ s^-1^). D) As in (A), but for the light intensity at which 95% maximum assimilation rate is achieved (PPFD _A95_, μmol m^−2^ s^−1^). E) As in (A), but for the light intensity at which light-saturated stomatal conductance is achieved (PPFD *_g_*_s sat_, μmol m^−2^ s^−1^). F) As in (A), but for the difference in irradiance between the light intensity at which 95% maximum assimilation rate and the light-saturated stomatal conductance is achieved (Δ PPFD, μmol m^-2^ s^-1^). All data represent an average across three independent single copy lines (*n* = 7 – 8 per individual line). Differences between transgenic plants and WT are assessed by Fisher LSD post-hoc analysis following a two-way ANOVA, where letters above each box represent statistically significant differences in mean values (p ≤ 0.05). The raw data can be found in Supplemental File 4.

## Supplemental File 1, Figure S7


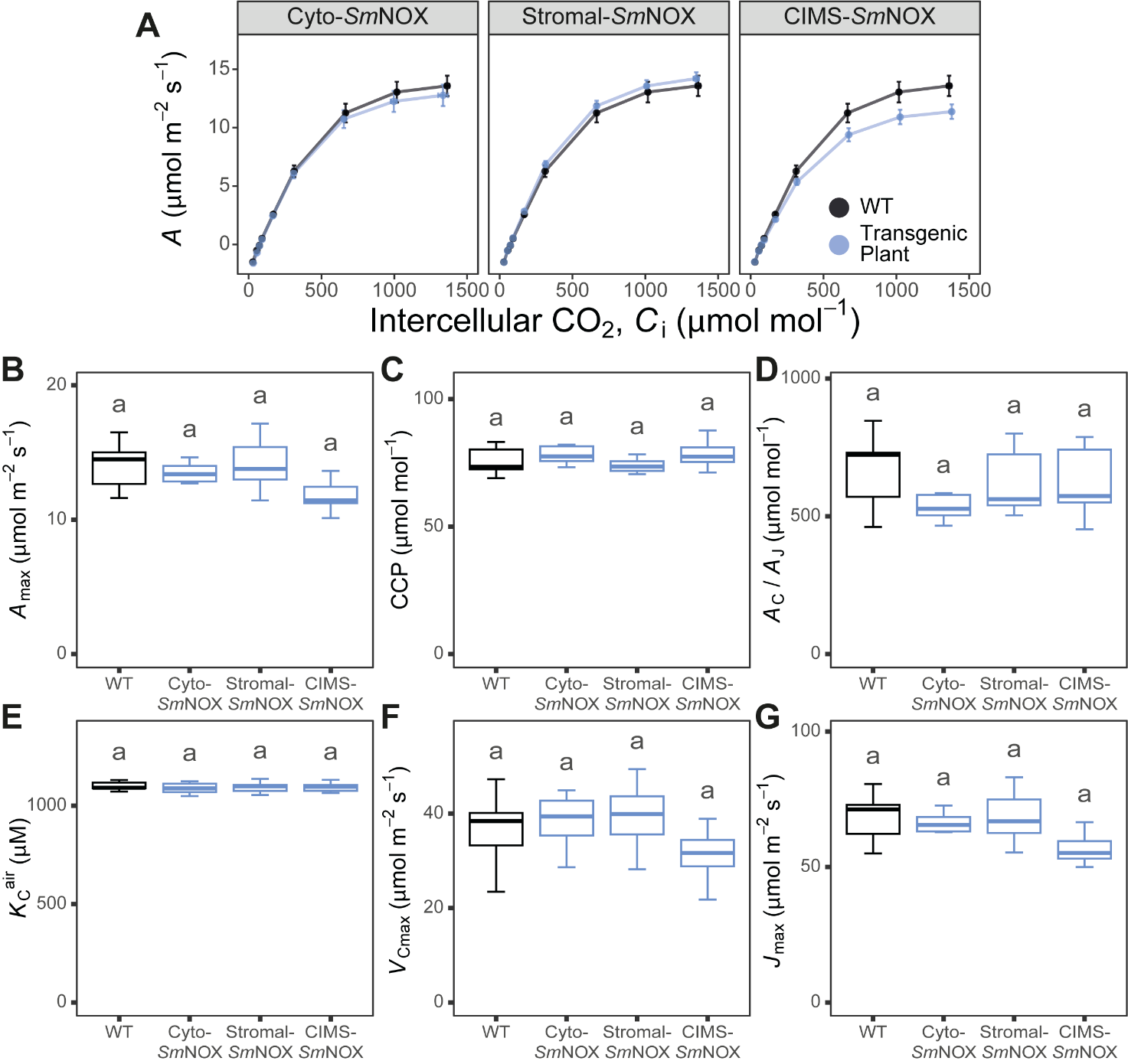


**Figure S7.** The response of plant gas exchange to intercellular CO_2_ concentration (*C*_i_, μmol mol). A) CO_2­_ assimilation rate (*A*, μmol m^−2^ s^−1^) at different *C*_i_ in wild-type (black) and transgenic plants (blue). WT: wild-type. Cyto-*Sm*NOX: cytosolic *Sm*NOX. Stromal-*Sm*NOX: chloroplast stromal *Sm*NOX. CIMS-*Sm*NOX: chloroplast intermembrane space *Sm*NOX. Data represent mean ± 1 S.E. B) Boxplot depicting the CO_2_–saturated assimilation rate (*A*_max_, μmol m^−2^ s^−1^). The colour scheme and abbreviations follow that described in (A). C) As in (B), but for the estimated CO_2_ compensation point (CCP, µmol mol^-1^). D) As in (B), but for the estimated CO_2_ concentration at the transition between rubisco-limited and ribulose-1,5-bisphosphate (RuBP) regeneration-limited photosynthesis (*A*_C_/*A*_J_, µmol mol^-1^). E) As in (B), but for the estimated Michaelis constant of rubisco for CO_2_ in 21% O_2_ air (*K*_C_^air^, µM). F) As in (B), but for the estimated maximum carboxylation rate of rubisco at 30°C (*V*_cmax_, µmol m^−2^ s^−1^). G) As in (B), but for the estimated maximum electron transport rate at 30°C (*J*_max_, µmol e^−^ m^−2^ s^−1^). All data represent an average across three independent single copy lines (*n* = 3 – 5 plants per line). Differences between transgenic plants and WT are assessed by Fisher LSD post-hoc analysis following a two-way ANOVA, where letters above each box represent statistically significant differences in mean values (p ≤ 0.05). The raw data can be found in Supplemental File 4.

## Supplemental File 1, Figure S8


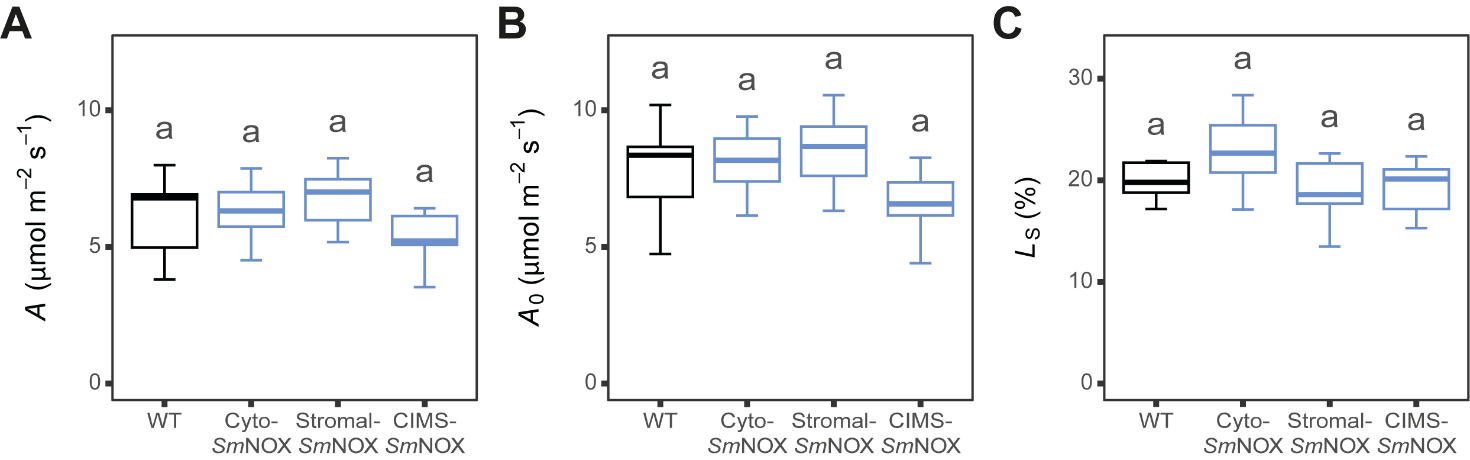


**Figure S8.** The stomatal limitation on plant photosynthesis estimated from CO_2_ response (*A* – *C*_i_) curves. A) Boxplot depicting the assimilation rate at ambient 400 μmol mol^-1^ CO_2_ (*A*, μmol m^−2^ s^−1^) in wild-type (black) and transgenic plants (blue). WT: wild-type. Cyto-*Sm*NOX: cytosolic *Sm*NOX. Stromal-*Sm*NOX: chloroplast stromal *Sm*NOX. CIMS-*Sm*NOX: chloroplast intermembrane space *Sm*NOX. B) As in (A), but for the assimilation rate which would occur at ambient CO_2_ if there was no stomatal resistance to CO_2_ diffusion (*A*_0_, μmol m^−2^ s^−1^). C) As in (A), but for the estimated stomatal limitation to assimilation at ambient 400 ppm CO_2_ (*L*_S_, %). Stomatal limitation is computed using the formula *L*_S_ = (*A*_0_ - *A*) / *A*_0_. All data represent an average across three independent single copy lines (*n* = 3 – 5 per individual line). Differences between transgenic plants and WT are assessed by Fisher LSD post-hoc analysis following a two-way ANOVA, where letters above each box represent statistically significant differences in mean values (p ≤ 0.05). The raw data can be found in Supplemental File 4.

## Supplemental File 1, Figure S9

**
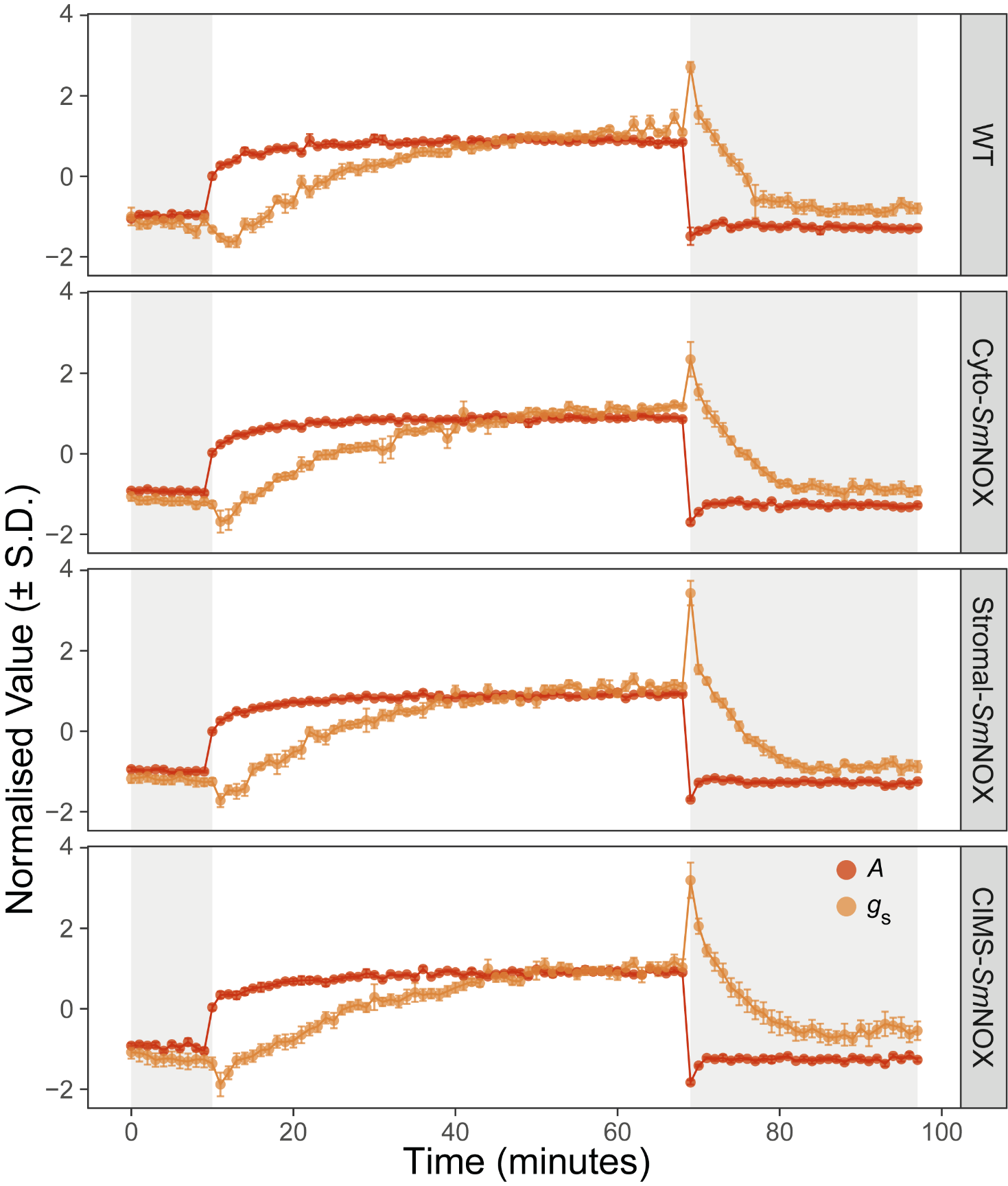
**

**Figure S9.** The normalised temporal response of plant CO_2­_ assimilation rate (*A*, μmol m^−2^ s^−1^; red) and stomatal conductance (*g*_s,_ mol m^−2^ s^−1^; orange) to step changes in light intensity. Shaded and unshaded areas represent periods of light intensity of 100 μmol m^−2^ s^−1^ and 1000 μmol m^−2^ s^−1^, respectively. WT: wild-type. Cyto-*Sm*NOX: cytosolic *Sm*NOX. Stromal-*Sm*NOX: chloroplast stromal *Sm*NOX. CIMS-*Sm*NOX: chloroplast intermembrane space *Sm*NOX. For each parameter, data are normalised by the standard deviation away from the mean calculated across the entire temporal response of that individual. Data represent mean ± 1 S.E of normalised values. All data represent an average across three independent single copy lines (*n* = 2 – 4 per individual line). The raw data can be found in Supplemental File 4.

## Supplemental File 1, Figure S10

**
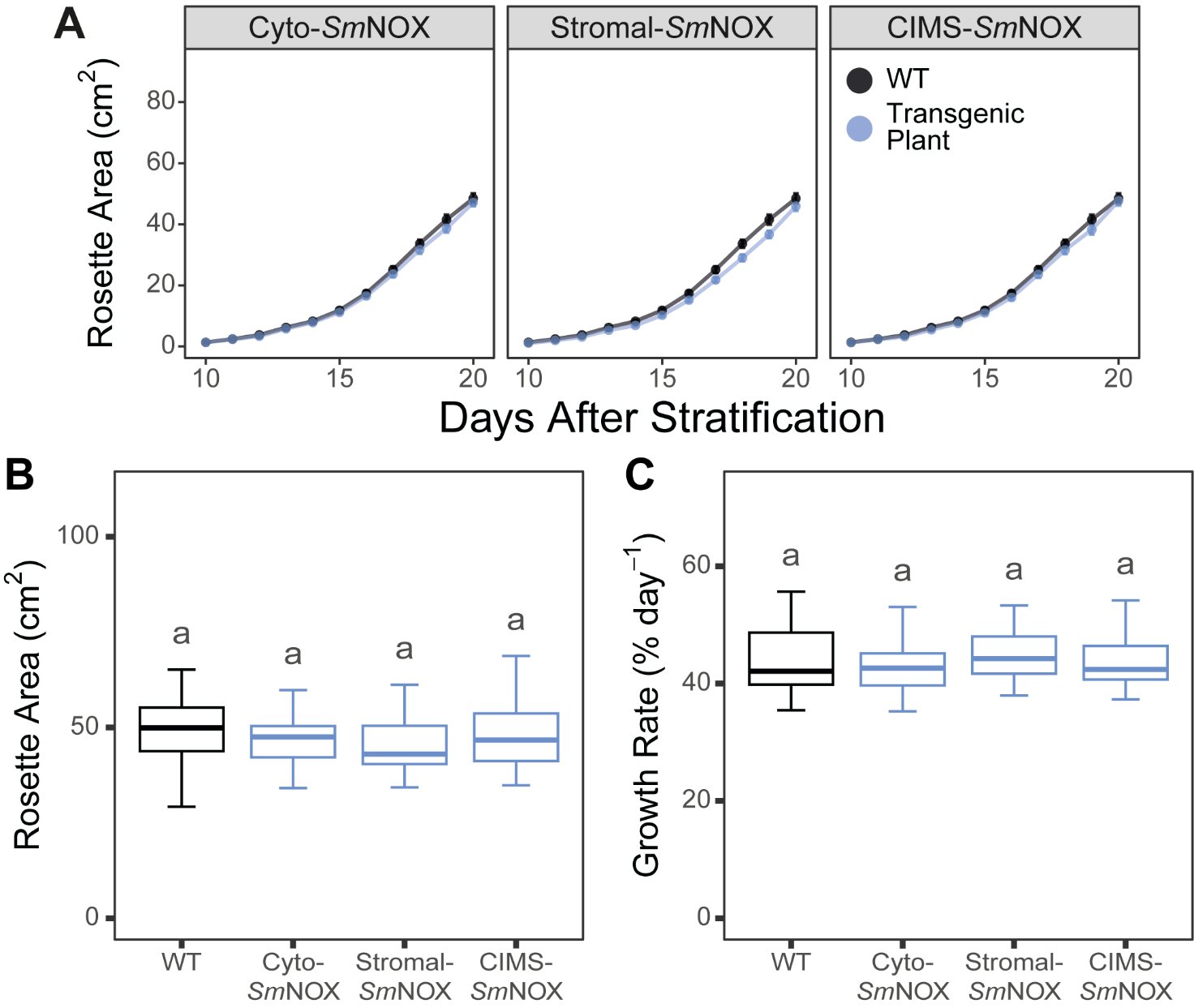
**

**Figure S10.** The vegetative and reproductive performance of plants under normal watering conditions. (A) The increase in visible rosette area (cm^2^) over time in wild-type (black) and transgenic plants (blue). WT: wild-type. Cyto-*Sm*NOX: cytosolic *Sm*NOX. Stromal-*Sm*NOX: chloroplast stromal *Sm*NOX. CIMS-*Sm*NOX: chloroplast intermembrane space *Sm*NOX. Data represent mean ± 1 S.E. B) Boxplot depicting the maximum visible rosette area at the end of vegetative growth (day = 20). The colour scheme and abbreviations follow that described in (A). C) As in (B), but for the average percentage growth rate of plants computed over the vegetative growth phase (% increase in rosette area day^-1^). All data represent an average across three independent single copy lines (*n* = 10 per individual line for all measurements). Differences between transgenic plants and WT are assessed by Fisher LSD post-hoc analysis following a two-way ANOVA, where letters above each box represent statistically significant differences in mean values (p ≤ 0.05). The raw data can be found in Supplemental File 4.

# Supplemental Tables

## Supplemental File 1, Table S1

**Table S1.** Predicted localisations of *Sm*NOX (including C-terminal GFP sequence) using TargetP V2.0 software ^15,16^.

|  | **Likelihood** | | | |
| --- | --- | --- | --- | --- |
|  | **Other** | **Mitochondrial TP** | **Chloroplast TP** | **Thylakoid TP** |
| **Cyto-*Sm*NOX** | 0.9948 | 0.0027 | 0 | 0 |

## Supplemental File 1, Table S2

**Table S2.** Gas exchange parameters derived from light response (*A* – *PPFD*_­_) curves. Values represent mean ± 1 S.E. All data represent an average across three independent single copy lines (*n* = 7 – 8 per individual line). Differences between transgenic plants and WT are assessed by Fisher LSD post-hoc analysis following a two-way ANOVA, where different letters represent statistically significant differences in mean values (p ≤ 0.05). Significant differences to WT are also highlighted in bold for ease of visualisation.

|  | **WT** | **Cyto-*Sm*NOX** | **Stromal-*Sm*NOX** | **CIMS-*Sm*NOX** |
| --- | --- | --- | --- | --- |
| ***A* _sat_**  (µmol m^−2^ s^−1^) | 6.2 ± 0.3 ^a^ | 5.3 ± 0.2 ^a^ | 5.8 ± 0.2 ^a^ | 5.2 ± 0.2 ^a^ |
| ***g*_s sat_**  (mol m⁻² s⁻¹) | 0.166 ± 0.008 ^a^ | **0.126 ± 0.006 ^b^** | **0.129 ± 0.005 ^b^** | **0.121 ± 0.006 ^b^** |
| ***iWUE* _sat_**  (µmol mol^-1^ H_2_O) | 41.3 ± 1.4 ^b^ | **46.5 ± 1.6 ^a^** | **48.5 ± 1.0 ^a^** | **48.2 ± 2.2 ^a^** |
| **LCP**  (µmol m^−2^ s^−1^) | 17.7 ± 1.7 ^a^ | 23.3 ± 1.7 ^a^ | 18.5 ± 1.7 ^a^ | 20.5 ± 1.3 ^a^ |
| ***R*_d_**  (µmol m^−2^ s^−1^) | 0.748 ± 0.084 ^a^ | 0.941 ± 0.075 ^a^ | 0.746 ± 0.074 ^a^ | 0.783 ± 0.048 ^a^ |
| ΦJ  (unitless) | 0.043 ± 0.002 ^a^ | 0.041 ± 0.002 ^a^ | 0.040 ± 0.001 ^a^ | 0.040 ± 0.002 ^a^ |
| ***J*_sat_**  (µmol m^−2^ s^−1^) | 59.2 ± 2.1 ^a^ | 57.7 ± 1.9 ^a^ | 57.8 ± 1.6 ^a^ | 54.0 ± 1.5 ^a^ |

***N.B.*** *A*_sat_: Light-saturated assimilation rate. *g*_s sat_: Light-saturated stomatal conductance. *iWUE*_sat_: Light-saturated intrinsic water-use efficiency. LCP: Light compensation point. R_d_: Mitochondrial respiration in the light. ΦJ: Modelled apparent quantum yield computed as the initial slope of the response of J to PPFD. *J*_sat_: Light-saturated electron transport rate.

## Supplemental File 1, Table S3

**Table S3.** Gas exchange parameters describing the relative coordination between plant photosynthesis and gas exchange derived from light response (*A* – *PPFD*_­_) curves. Values represent mean ± 1 S.E. All data represent an average across three independent single copy lines (*n* = 7 – 8 per individual line). Differences between transgenic plants and WT are assessed by Fisher LSD post-hoc analysis following a two-way ANOVA, where different letters represent statistically significant differences in mean values (p ≤ 0.05). Significant differences to WT are also highlighted in bold for ease of visualisation.

|  | **WT** | **Cyto-*Sm*NOX** | **Stromal-*Sm*NOX** | **CIMS-*Sm*NOX** |
| --- | --- | --- | --- | --- |
| ***g_s_* _A95_**  (mol m^−2^ s^−1^) | 0.154 ± 0.008 ^a^ | **0.120 ± 0.006 ^b^** | **0.125 ± 0.005 ^b^** | **0.116 ± 0.006 ^b^** |
| ***g*_s sat_**  (mol m^−2^ s^−1^) | 0.166 ± 0.008 ^a^ | **0.126 ± 0.006 ^b^** | **0.129 ± 0.005 ^b^** | **0.121 ± 0.006 ^b^** |
| **Δ *g_s_***  (mol m^−2^ s^−1^) | 0.012 ± 0.004 ^a^ | **0.005 ± 0.001 ^b^** | **0.004 ± 0.001 ^b^** | **0.005 ± 0.001 ^b^** |
| **PPFD _A95_**  (µmol m^−2^ s^−1^) | 471.4 ± 18.4 ^a^ | 695.2 ± 89.9 ^a^ | 581.0 ± 71.6 ^a^ | 642.9 ± 98.0 ^a^ |
| **PPFD *_g_*_s sat_**  (µmol m^−2^ s^−1^) | 1857.1 ± 245.8 ^a^ | 1523.8 ± 157.9 ^a^ | 1202.4 ± 160.2 ^a^ | 1490.5 ± 175.9 ^a^ |
| **Δ PPFD**  (µmol m^−2^ s^−1^) | 1385.7 ± 235.5 ^a^ | **828.6 ± 161.3 ^b^** | **621.4 ± 142.8 ^b^** | **847.6 ± 169.7 ^b^** |

***N.B.*** *g*_s_ _A95_: stomatal conductance at 95% maximum assimilation rate. *g_s sat_*: Light-saturated stomatal conductance. Δ *g*_s_: The difference in stomatal conductance at 95% maximum assimilation rate and the light-saturated stomatal conductance rate. PPFD _A95_: Light intensity at which 95% maximum assimilation rate is achieved. PPFD *_gs sat_*: Light intensity at which light-saturated stomatal conductance is achieved. Δ PPFD: The difference in irradiance between the light intensity at which 95% maximum assimilation rate is achieved and the light-saturated stomatal conductance is achieved.

## Supplemental File 1, Table S4

**Table S4.** Gas exchange parameters derived from CO_2_ response (*A* – *C*_i­_) curves. Values represent mean ± 1 S.E. All data represent an average across three independent single copy lines (*n* = 3 – 5 per individual line). Differences between transgenic plants and WT are assessed by Fisher LSD post-hoc analysis following a two-way ANOVA, where different letters represent statistically significant differences in mean values (p ≤ 0.05). Significant differences to WT are also highlighted in bold for ease of visualisation.

|  | **WT** | **Cyto-*Sm*NOX** | **Stromal-*Sm*NOX** | **CIMS-*Sm*NOX** |
| --- | --- | --- | --- | --- |
| ***A* _max_**  (µmol m^−2^ s^−1^) | 13.6 ± 0.9 ^a^ | 12.8 ± 0.9 ^a^ | 14.2 ± 0.5 ^a^ | 11.4 ± 0.6 ^a^ |
| **CCP**  (µmol mol^-1^) | 75.7 ± 1.6 ^a^ | 79.7 ± 2.2 ^a^ | 72.9 ± 1.4 ^a^ | 78.3 ± 1.6 ^a^ |
| ***K*_C_^air^**  (µM) | 1100.4 ± 6.9 ^a^ | 1089.2 ± 7.9 ^a^ | 1092.7 ± 6.9 ^a^ | 1093.8 ± 6.7 ^a^ |
| ***V*_c max_**  (µmol m^−2^ s^−1^) | 37.0 ± 2.6 ^a^ | 37.3 ± 2.4 ^a^ | 39.4 ± 1.9 ^a^ | 31.4 ± 1.5 ^a^ |
| ***V*_c max_ ^25°C^**  (µmol m^−2^ s^−1^) | 21.2 ± 1.6 ^a^ | 21.6 ± 1.4 ^a^ | 22.8 ± 1.1 ^a^ | 18.1 ± 0.9 ^a^ |
| ***J* _max_**  (µmol m^−2^ s^−1^) | 66.1 ± 4.4 ^a^ | 62.8 ± 4.5 ^a^ | 68.7 ± 2.7 ^a^ | 55.0 ± 3.1 ^a^ |
| ***J* _max_ ^25°C^**  (µmol m^−2^ s^−1^) | 54.7 ± 3.7 ^a^ | 52.0 ± 3.8 ^a^ | 56.9 ± 2.2 ^a^ | 45.6 ± 2.5 ^a^ |
| ***A*_C_/*A*_J_**  (µmol mol^-1^) | 657.6 ± 41.6 ^a^ | 563.3 ± 27.8 ^a^ | 622.7 ± 34.6 ^a^ | 626.4 ± 38.2 ^a^ |

***N.B.*** *A* _max_: CO_2_ saturated assimilation rate. CCP: CO_2_ compensation point. *K*_C_^air^: Modelled Michaelis constant of rubisco for CO_2_ in 21% O_2_ air. *V*_c max_: Modelled maximum carboxylation rate of rubisco at assay temperature of 30°C. *V*_c max_ ^25°C^: Modelled maximum carboxylation rate of rubisco adjusted to 25°C. *J* _max_: Maximum electron transport rate at assay temperature of 30°C. *J* _max_ ^25°C^: Maximum electron transport rate adjusted to 25°C. *A*_C_/*A*_J_: Modelled CO_2_ concentration at the transition between rubisco-limited and ribulose-1,5-bisphosphate (RuBP) regeneration-limited photosynthesis.

***Supplemental File 1, Table S5***

**Table S5.** The extent of stomatal limitation derived from CO_2_ response (*A* – *C*_i_) curves. Values represent mean ± 1 S.E. All data represent an average across three independent single copy lines (n = 3 – 5 per individual line). Differences between transgenic plants and WT are assessed by Fisher LSD post-hoc analysis following a two-way ANOVA, where different letters represent statistically significant differences in mean values (p ≤ 0.05). Significant differences to WT are also highlighted in bold for ease of visualisation.

|  | **WT** | **Cyto-*Sm*NOX** | **Stromal-*Sm*NOX** | **CIMS-*Sm*NOX** |
| --- | --- | --- | --- | --- |
| ***A***  (µmol m^−2^ s^−1^) | 6.3 ± 0.5 ^a^ | 6.1 ± 0.4 ^a^ | 6.8 ± 0.3 ^a^ | 5.4 ± 0.3 ^a^ |
| ***A*_0_**  (µmol m^−2^ s^−1^) | 7.9 ± 0.6 ^a^ | 7.9 ± 0.5 ^a^ | 8.5 ± 0.4 ^a^ | 6.6 ± 0.3 ^a^ |
| ***L*_S_**  (%) | 20.5 ± 1.0 ^a^ | 22.8 ± 1.0 ^a^ | 19.2 ± 0.8 ^a^ | 19.3 ± 0.8 ^a^ |

***N.B.*** *A*: CO_2_ assimilation rate at ambient CO_2_. *A*_0_: Predicted CO_2_ assimilation rate at ambient CO_2_ if there was no resistance to CO_2_ diffusion. *L*_S_: The estimated stomatal limitation to assimilation at ambient CO_2_ computed from *A* and *A*_0_.

## Supplemental File 1, Table S6

**Table S6.** Stomatal kinetic parameters derived from the temporal profiles of plant gas exchange responses to step changes in light intensity between 100 μmol m^−2^ s^−1^ and 1000 μmol m^−2^ s^−1^. Values represent mean ± 1 S.E. All data represent an average across three independent single copy lines (*n* = 3 – 4 per individual line). Differences between transgenic plants and WT are assessed by Fisher LSD post-hoc analysis following a two-way ANOVA, where different letters represent statistically significant differences in mean values (p ≤ 0.05). Significant differences to WT are also highlighted in bold for ease of visualisation.

|  | **WT** | **Cyto-*Sm*NOX** | **Stromal-*Sm*NOX** | **CIMS-*Sm*NOX** |
| --- | --- | --- | --- | --- |
| ***λ***  (minutes) | 1.8 ± 0.2 ^a^ | 1.2 ± 0.1 ^a^ | 1.4 ± 0.3 ^a^ | 1.6 ± 0.4 ^a^ |
| ***Sl* _max_**  (mol m^−2^ s^−1^ min^-1^) | 0.072 ± 0.008 ^a^ | 0.089 ± 0.011 ^a^ | 0.078 ± 0.011 ^a^ | 0.045 ± 0.004 ^a^ |
| ***k*_i_**  (minutes) | 10.0 ± 0.8 ^a^ | 8.5 ± 0.5 ^a^ | 8.4 ± 0.6 ^a^ | 10.5 ± 1.1 ^a^ |
| ***k*_d_** (minutes) | 2.1 ± 0.3 ^a^ | 2.6 ± 0.3 ^a^ | 2.1 ± 0.2 ^a^ | 2.0 ± 0.3 ^a^ |

***N.B.*** λ: Initial lag in response time of stomatal conductance upon step increase in light intensity from 100 μmol m^−2^ s^−1^ to 1000 μmol m^−2^ s^−1^. *Sl* _max_: Maximal rate of stomatal opening upon step increase in light intensity from 100 μmol m^−2^ s^−1^ to 1000 μmol m^−2^ s^−1^ min^-1^. *k*_i_: Time taken to achieve new steady state stomatal conductance upon step increase in light intensity from 100 μmol m^−2^ s^−1^ to 1000 μmol m^−2^ s^−1^. *k*_d_: Time taken to achieve new steady state stomatal conductance upon step decrease in light intensity from 1000 μmol m^−2^ s^−1^ to 100 μmol m^−2^ s^−1^.

# References

1. Engler, C., Gruetzner, R., Kandzia, R. & Marillonnet, S. Golden gate shuffling: A one-pot DNA shuffling method based on type ils restriction enzymes. *PLoS One* **4**, (2009).

2. Gantner, J. *et al.* Peripheral infrastructure vectors and an extended set of plant parts for the Modular Cloning system. *PLoS One* **13**, (2018).

3. Engler, C. *et al.* A Golden Gate modular cloning toolbox for plants. *ACS Synth. Biol.* **3**, 839–843 (2014).

4. Weber, E., Engler, C., Gruetzner, R., Werner, S. & Marillonnet, S. A modular cloning system for standardized assembly of multigene constructs. *PLoS One* **6**, (2011).

5. Grützner, R. *et al.* High-efficiency genome editing in plants mediated by a Cas9 gene containing multiple introns. *Plant Commun.* **2**, (2021).

6. Marshall, B. & Biscoe, P. V. A model for C3 leaves describing the dependence of net photosynthesis on irradiance. *J. Exp. Bot.* **31**, 29–39 (1980).

7. Stinziano, J. R. *et al.* Photosynthesis: tools for plant ecophysiology & modeling. *R package version* vol. 2 at (2020).

8. Stinziano, J. R. *et al.* Principles of resilient coding for plant ecophysiologists. *AoB Plants* **13**, (2021).

9. Duursma, R. A. Plantecophys - An R package for analysing and modelling leaf gas exchange data. *PLoS One* **10**, (2015).

10. Farquhar, G. D. & Sharkey, T. D. Stomatal Conductance and Photosynthesis. *Annu. Rev. Plant Physiol.* **33**, 317–345 (1982).

11. Vialet-Chabrand, S., Dreyer, E. & Brendel, O. Performance of a new dynamic model for predicting diurnal time courses of stomatal conductance at the leaf level. *Plant, Cell Environ.* **36**, 1529–1546 (2013).

12. McAusland, L. *et al.* Effects of kinetics of light-induced stomatal responses on photosynthesis and water-use efficiency. *New Phytol.* **211**, 1209–1220 (2016).

13. Adrian, J. *et al.* Transcriptome dynamics of the stomatal lineage: Birth, amplification, and termination of a self-renewing population. *Dev. Cell* **33**, 107–118 (2015).

14. Woo, H. R. *et al.* Programming of plant leaf senescence with temporal and inter-organellar coordination of transcriptome in arabidopsis. *Plant Physiol.* **171**, 452–467 (2016).

15. Emanuelsson, O., Nielsen, H., Brunak, S. & Von Heijne, G. Predicting subcellular localization of proteins based on their N-terminal amino acid sequence. *J. Mol. Biol.* **300**, 1005–1016 (2000).

16. Armenteros, J. J. A. *et al.* Detecting sequence signals in targeting peptides using deep learning. *Life Sci. Alliance* **2**, (2019).
